# Alterations in cytoskeletal and Ca^2+^ cycling regulators in atria lacking the obscurin Ig58/59 module

**DOI:** 10.3389/fcvm.2023.1085840

**Published:** 2023-04-13

**Authors:** Alyssa Grogan, Weiliang Huang, Annie Brong, Maureen A. Kane, Aikaterini Kontrogianni-Konstantopoulos

**Affiliations:** ^1^Department of Biochemistry and Molecular Biology, University of Maryland, Baltimore, MD, United States; ^2^Department of Pharmaceutical Sciences, University of Maryland School of Pharmacy, Baltimore, MD, United States

**Keywords:** obscurin, atrial fibrillation, arrhythmia, Z-disk, Ca^2+^ cycling, cytoskeleton

## Abstract

**Introduction:**

Obscurin (720–870 kDa) is a giant cytoskeletal and signaling protein that possesses both structural and regulatory functions in striated muscles. Immunoglobulin domains 58/59 (Ig58/59) of obscurin bind to a diverse set of proteins that are essential for the proper structure and function of the heart, including giant titin, novex-3, and phospholamban (PLN). Importantly, the pathophysiological significance of the Ig58/59 module has been further underscored by the discovery of several mutations within Ig58/59 that are linked to various forms of myopathy in humans. We previously generated a constitutive deletion mouse model, *Obscn*-*ΔIg58/59*, that expresses obscurin lacking Ig58/59, and characterized the effects of this deletion on cardiac morphology and function through aging. Our findings demonstrated that *Obscn*-*ΔIg58/59* male animals develop severe arrhythmia, primarily manifesting as episodes of junctional escape and spontaneous loss of regular p-waves, reminiscent of human atrial fibrillation, accompanied by significant atrial enlargement that progresses in severity with aging.

**Methods and Results:**

To comprehensively characterize the molecular alterations responsible for these pathologies, we performed proteomic and phospho-proteomic analyses in aging *Obscn*-*ΔIg58/59* atria. Our studies revealed extensive and novel alterations in the expression and phosphorylation profile of major cytoskeletal proteins, Ca^2+^ regulators, and Z-disk associated protein complexes in the *Obscn*-*ΔIg58/59* atria through aging.

**Discussion:**

These studies implicate obscurin, particularly the Ig58/59 module, as an essential regulator of the Z-disk associated cytoskeleton and Ca^2+^ cycling in the atria and provide new molecular insights into the development of atrial fibrillation and remodeling.

## Introduction

Obscurin (720–870 kDa), comprised of 65–67 tandemly arranged immunoglobulin (Ig) domains, 2–3 fibronectin-III motifs, and a unique assortment of signaling domains at its COOH-terminus depending on the isoform, is a giant cytoskeletal protein that localizes to the periphery of M-bands and Z-disks where it modulates diverse structural and regulatory functions in striated muscles (1, 2). Given its large size, modular nature, and unique cellular distribution as a peripheral sarcomeric protein, obscurin is ideally situated to interact with proteins localizing to different cellular compartments, ranging from the sarcomere and the surrounding sarcoplasmic reticulum (SR) membranes to the cytoskeleton and the sarcolemma (1, 2). Accordingly, obscurin serves essential roles in the assembly and stabilization of the myofibril, Ca^2+^ signaling, cell adhesion, and the physical integration of the sarcomere with the cytoskeleton and surrounding membrane structures (1, 2).

Over the past several decades, the discovery of >20 missense, splicing, and frameshift mutations spanning the entire length of the obscurin gene (*OBSCN*) in patients with hypertrophic cardiomyopathy (HCM), dilated cardiomyopathy (DCM), left ventricular non-compaction (LVNC), and arrhythmogenic right ventricular cardiomyopathy (ARVC) has increasingly implicated obscurin in the development of cardiac disease in humans (3–5). To date, the disease mechanisms underlying the majority of these mutations have remained entirely uninvestigated, with the exception of the HCM-linked R4344Q variant residing within obscurin Ig58 which our lab has previously characterized (6). Our findings demonstrated that mice carrying the R4344Q variant (*Obscn-R4344Q*) exhibited a “gain-of-function” phenotype wherein enhanced binding between mutant Ig58 and phospholamban (PLN) resulted in disinhibition of the sarco-endoplasmic reticulum Ca^2+^ ATPase (SERCA), increased Ca^2+^ cycling kinetics, and the development of ventricular arrhythmia through aging (6). The direct binding between obscurin-Ig58/59 and PLN and their enhanced association in the presence of the R4344Q variant was recently corroborated by Fukuzawa and colleagues, reporting a ~ 2.5-fold decrease in the K_d_; yet, the physiological relevance of the obscurin/PLN interaction was questioned (7). As a small modulatory protein that is extensively regulated by phosphorylation and assumes multiple oligomeric conformations in physiological settings, PLN inherently interacts weakly and/or transiently with its binding partners. Thus, technical limitations of different *in vitro* systems perhaps mask a complex and dynamically regulated (i.e., on a beat-to-beat basis) interaction between obscurin-Ig58/59 and PLN.

In addition to its binding to PLN, obscurin-Ig58/59 has been reported to interact with the extreme NH_2_-terminus of titin (3–4 MDa) as well as a unique 198-amino acid long sequence of titin’s smaller splice variant, novex-3 (~700 kDa), at the level of the Z-disk (8, 9). Of note, the obscurin/novex-3 interaction was recently contested by Fukuzawa and colleagues (7). Nevertheless, given that obscurin-Ig58/59 may interact with a diverse set of structural and regulatory proteins that are essential for normal muscle function, we generated the *Obscn-ΔIg58/59* model that expresses obscurin constitutively lacking Ig58/59 to extensively characterize the pathophysiological significance of this region in the heart (10). Our studies demonstrated that sedentary *Obscn-ΔIg58/59* males develop severe arrhythmia characterized by frequent episodes of spontaneous junctional escape and atrial fibrillation beginning at 6-months of age accompanied by significantly increased atrial mass and dilated left ventricles by 12-months (10).

Herein, we performed proteomic and phospho-proteomic analysis using 6- and 12-month old *Obscn-ΔIg58/59* atria in order to comprehensively investigate the molecular basis for the prominent atrial arrhythmia and remodeling in aging *Obscn-ΔIg58/59* males. Our studies revealed extensive and novel changes in the expression and phosphorylation profile of the *Obscn-ΔIg58/59* atrial proteome, mainly impacting cytoskeletal and signaling complexes at the Z-disk and Ca^2+^ regulating proteins. Together, these results provide new molecular insights into the pathophysiology of spontaneous atrial arrhythmia and remodeling.

## Materials and methods

### *Obscn-ΔIg58/59* constitutive deletion mice

The *Obscn-ΔIg58/59* constitutive deletion model was generated and genotyped as previously described (10). Animal care and procedures were conducted under protocols approved by the Institutional Animal Care and Use Committee at the University of Maryland, School of Medicine (UMSOM) and in accordance with the NIH guidelines (Guide for the Care and Use of Laboratory Animals).

### Lysate preparation and western blotting

**Figure 1:**
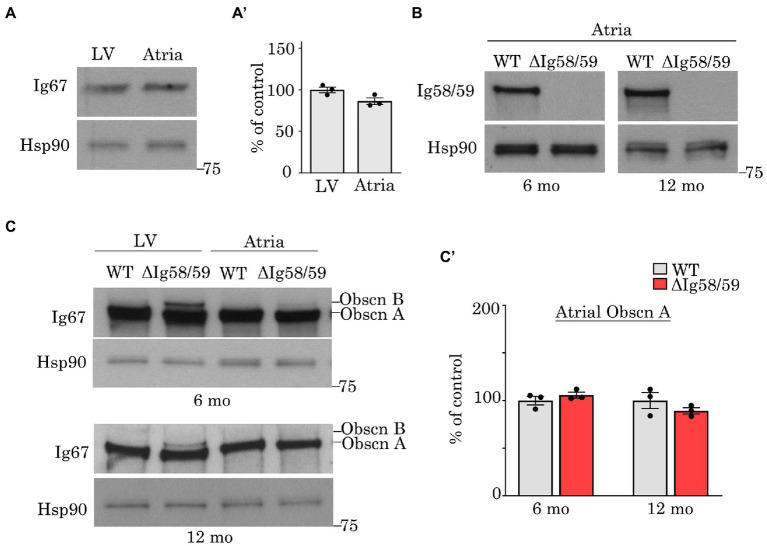
Expression levels of obscurin in wild-type and *Obscn-ΔIg58/59* atria. **(A,A’)** Immunoblotting **(A)** and relative quantification **(A’)** of giant obscurin in wild-type 12-month-old left ventricles (LV) and atrial lysates utilizing antibodies to Ig67 did not reveal significant expression differences; *t*-test, *p* = 0.18. **(B)** Immunoblotting of giant obscurin in 6- and 12-month-old wild-type and *Obscn-ΔIg5859* atria using antibodies to Ig58/59 confirmed the Ig58/59 deletion. **(C,C’)** Immunoblotting **(C)** and relative quantification **(C’)** of giant obscurin in 6- and 12-month-old wild-type and *Obscn-ΔIg5859* LV and atria using antibodies to Ig67 indicated that obscurin A expression is unchanged in *Obscn-ΔIg5859* atria compared to wild-type; *t*-test, *p* = 0.32 (6-months), *p* = 0.36 (12-months). Notably, the up-regulation of obscurin B observed in *Obscn-ΔIg5859* LV was barely detectable in *Obscn-ΔIg5859* atria; *n* = 3 animals per group; data points represent the average of at least two technical replicas; densitometric values were normalized to Hsp90, which was used as loading control.

**Figure 2:**
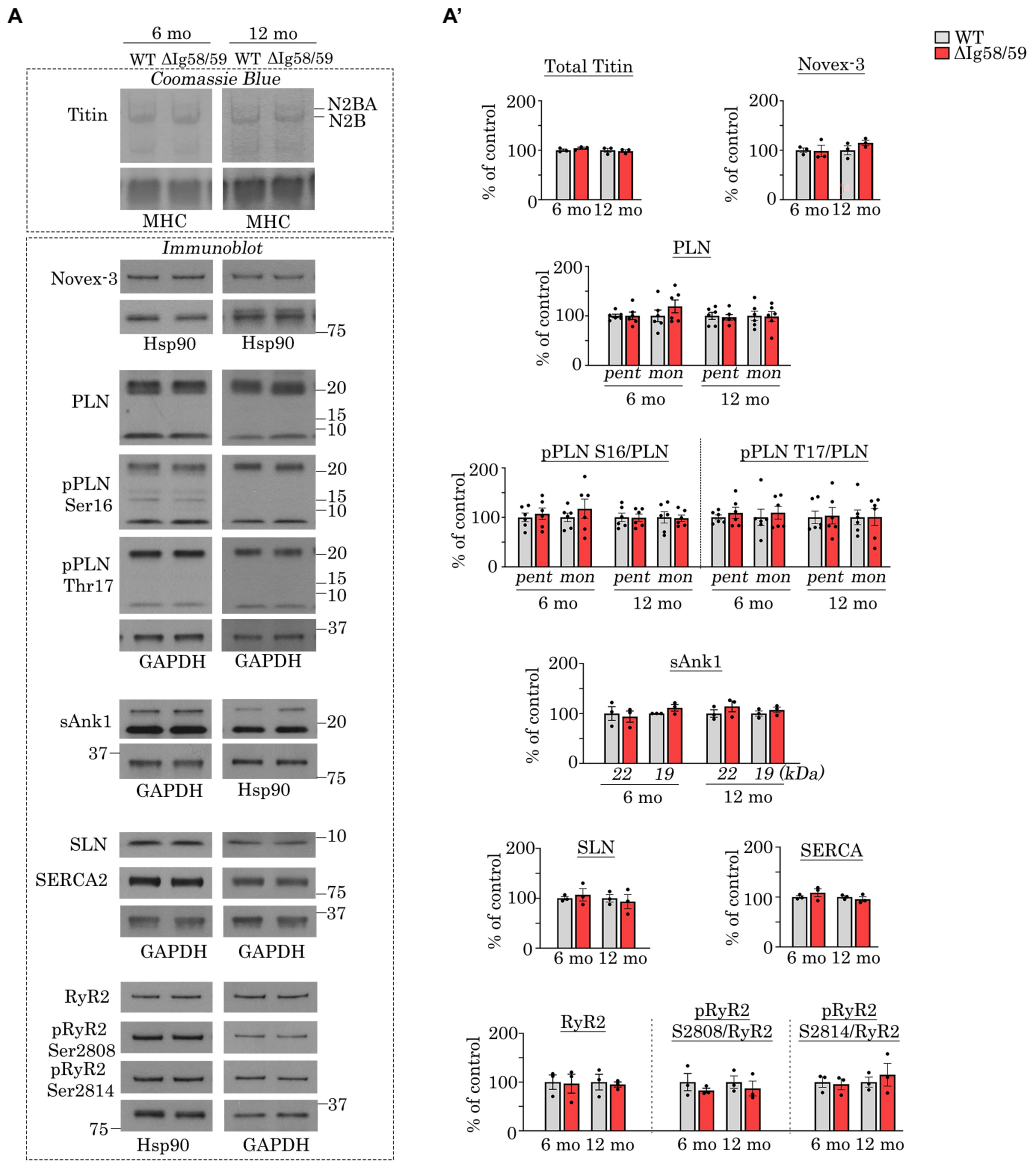
Expression and phosphorylation status of Ig58/59 binding partners and selected Ca^2+^ cycling proteins are unaffected in aged *Obscn-ΔIg58/59* atria. **(A,A’)** Representative Coomassie Blue stained agarose gels and immunoblots **(A)** and relative quantifications **(A’)** did not reveal statistically significant alterations in the expression or phosphorylation status of giant titins, novex-3, PLN, sAnk1, SLN, SERCA2, or RyR2 in lysates prepared from 6- and 12-month-old *Obscn-ΔIg5859* atria; MHC, Hsp90, and GAPDH served as loading controls; *n* = 3–6 animals per group; data points represent the average of at least two technical replicas; quantification of phosphorylation levels are normalized to total PLN or RyR2 levels; pent, pentamer; mono, monomer.

Lysates were prepared from flash frozen cardiac tissue and protein expression was evaluated by immunoblotting as previously described (10). Briefly, frozen right and left atria were combined, ground to a powder in a glass homogenizer while immersed in liquid nitrogen, and incubated at −20°C for 20 min. The ground tissue was solubilized in urea-thiourea lysis buffer (8 mol/L urea, 2 mol/L thiourea, 3% SDS, 0.05 mol/L tris–HCl, 0.03% bromophenol blue, 0.075 mol/L dithiothreitol, pH 6.8) and 50% glycerol supplemented with protease and phosphatase inhibitors (Halt Protease and Phosphatase Inhibitor Cocktail, Thermo Fisher Scientific, Waltham, MA, United States) in a 60°C water bath. Homogenates were centrifuged and supernatants were aliquoted and flash frozen in liquid nitrogen. For western blotting, equal amounts of protein lysates were thawed at 55°C for 5 min, separated by SDS-polyacrylamide gel electrophoresis, transferred to nitrocellulose membrane, and probed with the respective primary antibodies. Alkaline phosphatase (AP)-conjugated or horseradish peroxidase (HRP)-conjugated secondary antibodies and the respective chemiluminescent reagents (NovaBright; AP, or Pierce ECL; HRP) were used to detect immunoreactive bands. Densitometry was performed using ImageJ and each band was normalized to a loading control (glyceraldehyde 3-phosphate dehydrogenase, GAPDH; heat shock protein 90, Hsp90; or α-actinin). At least two technical replicates of at least three different biological samples (i.e., hearts) were quantified per genotype for each protein evaluated. The original representative blots shown in Figures 1, 2 and 8 are included in Supplementary Image 1; please note that in some instances, immunoblots were flipped for ease of presentation.

**Figure 3:**
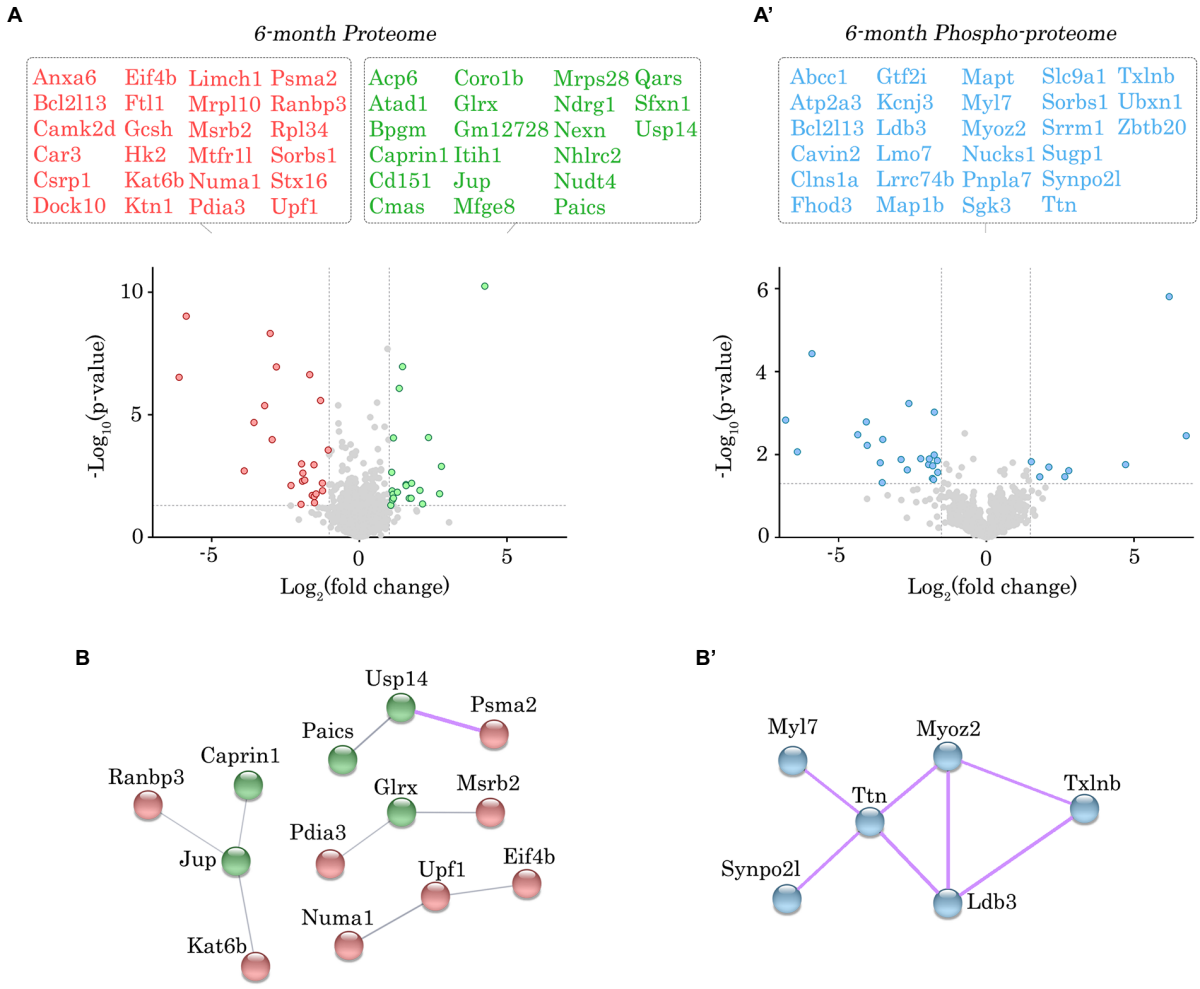
Proteomic and phospho-proteomic analysis of *Obscn-ΔIg58/59* atria at 6-months. **(A,A’)** Volcano plots of significantly up-regulated (green) or down-regulated (red) proteins **(A)** and significantly altered phospho-peptides (blue; **A’**) in *Obscn-ΔIg58/59* atria at 6-months. A total of 45 proteins out of 1700 detected exhibited significantly altered expression **(A)**, whereas 30 phospho-peptides out of 1,401 detected were significantly altered **(A’)** in 6-month-old *Obscn-ΔIg58/59* atria compared to wild-type; *n* = 5 biological samples per genotype; grey dotted lines represent thresholds of *p* < 0.05 and SD > 2. **(B,B’)** The physical and functional associations of the deregulated proteins **(B)** and phospho-proteins **(B’)** in *Obscn-ΔIg58/59* atria at 6-months were plotted using the STRING database (v.11.0b). Line thickness corresponds to the strength of the association. Protein networks with a high confidence score (>0.7) are highlighted in purple.

### Antibodies

The following primary antibodies were used for western blotting: rabbit polyclonal antibodies to obscurin Ig58/59 (1 μg/mL) (11), obscurin Ig67 (1 μg/mL) (10), novex-3 (1:1000, a generous gift from Dr. Henk Granzier) (9), PLN-pSer16 (1:1000; 07–052, Millipore, Temecula, CA, United States), PLN-pThr17 (1:2000; A010-13AP, Badrilla, Leeds, United Kingdom), sAnk1 (1 μg/mL) (12), RyR2-pSer2808 (1:2000; ab59225, Abcam, Cambridge, MA, United States), RyR2-pSer2814 (1:500; A010-31AP, Badrilla), sarcolipin (1 μg/mL, a generous gift from Dr. Robert Bloch) (13), ERK2 (1:1000; CST-9108S, Cell Signaling Technology, Danvers, MA, United States), ERK1/2-pThr183/pTyr185 (1:2000; CST-4370 T, Cell Signaling Technology), rabbit monoclonal antibodies to Hsp90 (1:1000; CST-4877, Cell Signaling Technology), and mouse monoclonal antibodies to PLN (1:5000; ab2865, Abcam), SERCA2 (1:1000; MA3-919, Thermo Fisher Scientific, Waltham, MA, United States), RyR2 (1:1000; MA3-925, Thermo Fisher Scientific), GAPDH (1:15000; G8795, Millipore), and α-actinin (1:2500; A7811, Sigma-Aldrich, St. Louis, MO, United States). The following secondary antibodies were used for western blotting: goat anti-mouse IgG (1:3000; A3688, Sigma-Aldrich), goat anti-rabbit IgG (1:3000; AB_2337947, Jackson Immunoresearch, West Grove, PA, United States), goat anti-mouse IgG (1,3,000; CST-7076S, Cell Signaling Technology), and goat anti-rabbit IgG (1,3,000; CST-7074S, Cell Signaling Technology).

### Electrophoresis and Coomassie Blue staining for titin

Atrial lysates prepared as described above were separated on 16 × 18 cm gels composed of 1% agarose in 1X running buffer (50 mM tris, 0.384 mol/l glycine, 0.1% SDS) and 30% glycerol using the Hoefer SE600 unit system at 4°C for 3 h as described previously (10). Gels were stained with Coomassie Blue and the bands corresponding to giant titin were quantified using ImageJ and normalized to myosin heavy chain (MHC) as a loading control. Any digital adjustments to promote visualization of the bands were applied uniformly across the entire gel. At least two technical replicates of three different biological samples (i.e., hearts) were quantified for each genotype. The original representative titin gels shown in Figure 2 are included in Supplementary Image 1; please note that in some instances, gels were flipped for ease of presentation.

### Proteomic and phospho-proteomic analysis

Proteomic experiments were performed in the Mass Spectrometry Center at the University of Maryland School of Pharmacy. Atrial tissues dissected from male wild-type and homozygous *Obscn-ΔIg58/59* mice (*n* = 5 hearts per group) were homogenized in phosphate buffered saline using the Precellys CK14 lysing kit (Bertin Corp., Rockville, MD, United States). Proteins were extracted and purified from tissue lysates by trichloroacetic acid precipitation. Protein concentration was measured by bicinchoninic acid assay as described previously (14). Lysates were reduced, alkylated, and trypsinolyzed on a 10 K filter for shotgun proteomics as in (15). Phospho-peptides were enriched by TiO_2_ affinity chromatography (Sigma). Tryptic peptides were separated by a nanoACQUITY UPLC analytical column on a Waters nano-ACQUITY UPLC system and analyzed with a coupled Thermo Scientific Orbitrap Fusion Lumos Tribrid mass spectrometer as previously described (16). In detail, tryptic peptides were separated on a nano-ACQUITY UPLC analytical column (BEH130 C18, 1.7 μm, 75 μm × 200 mm, Waters) over a 165-min linear acetonitrile gradient (3–40%) with 0.1% formic acid on a Waters nano-ACQUITY UPLC system and analyzed on a coupled Thermo Scientific Orbitrap Fusion Lumos Tribrid mass spectrometer as previously reported (17). Full scans were acquired at a resolution of 120,000 and precursors were selected for fragmentation by higher-energy collisional dissociation (normalized collision energy at 30%) for a maximum 3-s cycle. Tandem mass spectra were searched against a UniProt reference *Mus musculus* proteome using Sequest HT algorithm (18) and MS Amanda algorithm (19) with a maximum precursor mass error tolerance of 10 ppm. Carbamidomethylation of cysteine was treated as static modification. Phosphorylation of serine (Ser), threonine (Thr), and tyrosine (Tyr), and deamidation of asparagine and glutamine were treated as dynamic modifications. Resulting hits were validated at a maximum global false discovery rate (FDR) of 0.01 using a semi-supervised machine learning algorithm Percolator (20). Label-free quantifications were performed using Minora, an aligned AMRT (Accurate Mass and Retention Time) cluster quantification algorithm (Thermo Fisher Scientific, 2017). Label-free quantitation of protein abundances was measured by comparing the MS1 peak volumes of peptide ions, whose identities were confirmed by MS2 sequencing.

The above abundance values were imported into Partek GS software for further statistical and bioinformatic analyses (Transcriptomics and Deep Sequencing Core, Johns Hopkins University). There were two mappings of the mass spectra: (a) to individual proteins, and (b) to unique phospho-peptides, wherein a phospho-peptide represents a unique specific position within its peptide. Both the individual proteins and unique phospho-peptide proteins were annotated with their cognate genes’ approved MGI/NCBI nomenclature. Following log_2_ transformation, the abundance values were then quantile normalized for each time point to minimize experimental noise among the lanes that represent replicate samples for the two biological classes (i.e., genotypes), and the *Obscn-ΔIg58/59* samples were compared to the wild type with two-tailed one-way *t*-test ANOVA. Each protein or unique phospho-peptide compared received a relative abundance and statistical value, as a fold-change and value of *p*, and the log_2_ fold changes were analyzed to determine their standard deviation from the mean value of no change. Proteins and phospho-peptides with a value of *p* of <0.05 and log_2_ fold changes that differed by >2 standard deviations were deemed to be significantly different. The precise phospho-site residues that exhibited a probability >75% are specified, whereas ambiguous phospho-site residues that displayed <75% probability are denoted in the associated phospho-peptide when referenced in the text and tables. Phosphorylation sites not previously annotated in PhosphoSitePlus (v. 6.6.0.4.) were denoted as novel. The raw mass spectrometry proteomics data have been deposited to the ProteomeXchange Consortium *via* the PRIDE (21) partner repository with the dataset identifier PXD028904. A complete list of all proteins and phospho-peptides that were identified at 6- and 12-months are listed with associated statistics in Supplementary Data Sheets S1–S2 and S3–S4, respectively.

### STRING analysis

The physical and functional associations of the proteins and phospho-proteins deemed to be significantly different between genotypes were plotted using the STRING database (v.11.0b) (22). To be as inclusive as possible, protein–protein interactions with a confidence score of >0.4 (i.e., medium confidence threshold) were plotted for differentially regulated proteins, while those relationships exhibiting high confidence scores (>0.7) were highlighted within the network. Only high confidence interactions (>0.7) were plotted and highlighted for deregulated phospho-proteins. Disconnected nodes and clusters comprised of less than 3 proteins were excluded.

### Enrichment analysis

The differentially expressed proteins and phospho-proteins were further analyzed with the QIAGEN Ingenuity Pathway Analysis (IPA) platform to determine their biological significance. Due to the large number of significantly altered pathways identified, the top 10 most significant pathways and cellular functions that are relevant to cardiovascular physiology and associated with at least 3 deregulated proteins/phospho-proteins were included in the text, figures, and tables. A complete list of all significantly altered pathways and cellular functions identified at 6- and 12-months are listed in Supplementary Data Sheets S5–S6 and S7–S8, respectively.

## Results and discussion

### Immunoblot analysis of aging *Obscn-ΔIg58/59* atria did not reveal significant differences in the levels of obscurin, titin, or canonical Ca^2+^ cycling proteins

We recently generated a constitutive deletion mouse model, *Obscn-ΔIg58/59*, that expresses obscurin lacking the Ig58/59 region and comprehensively evaluated the effects of this deletion on cardiac morphology and function through aging. Our studies showed that male *Obscn-ΔIg58/59* mice exhibit episodes of severe atrial arrhythmia by 6-months, manifesting as junctional escape and spontaneous loss of regular *p*-waves (10). By 12-months, the incidence and severity of arrhythmias intensified accompanied by significant atrial enlargement (10). Notably, female *Obscn-ΔIg58/59* mice do not exhibit any structural or functional deficiencies through aging and develop only mild arrhythmia that occurs less frequently compared to *Obscn-ΔIg58/59* males (10). Therefore, we focus our molecular characterization on *Obscn-ΔIg58/59* males only.

To investigate the mechanistic basis for the development of atrial fibrillation and remodeling in aging *Obscn-ΔIg58/59* males, we first evaluated the expression levels of obscurin, the binding partners of obscurin-Ig58/59, and a panel of Ca^2+^ regulators that are commonly associated with the development of atrial fibrillation. Earlier studies assessing the expression of obscurin during embryonic development reported reduced obscurin transcript levels in mouse atria compared to ventricles at embryonic day 12 (23). However, comparison of obscurin expression between adult atrial and ventricular tissues has not yet been experimentally determined. We therefore performed immunoblotting experiments using lysates prepared from 12-month-old wild-type hearts but did not observe significant differences in the levels of giant obscurin between the left ventricle and atria (Figures 1A,A’).

We next evaluated the impact of the Ig58/59 deletion on atrial obscurin expression in sedentary aging animals. Immunoblotting experiments utilizing antibodies to obscurin-Ig58/59 confirmed the absence of this region in lysates generated from 6- and 12-month-old *Obscn-ΔIg58/59* atria (Figure 1B). Similar to our prior findings in *Obscn-ΔIg58/59* left ventricles (10), antibodies to obscurin-Ig67 did not reveal significant differences in prototypical obscurin A expression between wild-type and *Obscn-ΔIg58/59* atria at 6- or 12-months (Figures 1C,C’). Intriguingly, the up-regulation of obscurin B (the largest known isoform containing two serine/threonine, Ser/Thr, kinases) previously reported in *Obscn-ΔIg58/59* left ventricular tissue (10) was barely detectable in *Obscn-ΔIg58/59* atria (Figures 1C,C’). This finding indicated that the Ig58/59 deletion leads to distinct molecular alterations in *Obscn-ΔIg58/59* atria compared to the ventricle and suggested that *Obscn-ΔIg58/59* atria potentially lack compensatory signaling mechanisms that could be mediated by obscurin-kinase bearing isoforms in *Obscn-ΔIg58/59* left ventricles.

We next evaluated the expression and phosphorylation levels of the known binding partners of Ig58/59 in addition to select Ca^2+^ cycling regulators. Interestingly, there were no statistically significant differences in the levels of giant titin, novex-3, PLN or its phosphorylation at Ser16 or Thr17, small ankyrin 1 (sAnk1), sarcolipin (SLN), SERCA2, RyR2 or its phosphorylation at Ser2808 or Ser2814 in *Obscn-ΔIg58/59* atria compared to age-matched controls at either 6- or 12-months (Figures 2A,A’). Therefore, the atrial remodeling and arrhythmia in aging *Obscn-ΔIg58/59* male mice cannot be explained by changes in the expression levels and/or canonical phosphorylation sites of these proteins, as is the case for *Obscn-ΔIg58/59* left ventricles (10). This suggested alternative mechanisms in the atria, perhaps involving additional Ca^2+^ or cytoskeletal regulators and/or less characterized/novel phosphorylation events. Along these lines, many studies have reported the presence of phosphorylation sites on titin (24), RyR2 (25–27), PLN (28), sAnk1 (29), and SERCA2 (30), for which their (patho)physiological impact has not been established.

### Proteomic and phospho-proteomic analysis revealed deregulated structural and regulatory proteins in aging *Obscn-ΔIg58/59* atria

**Figure 4:**
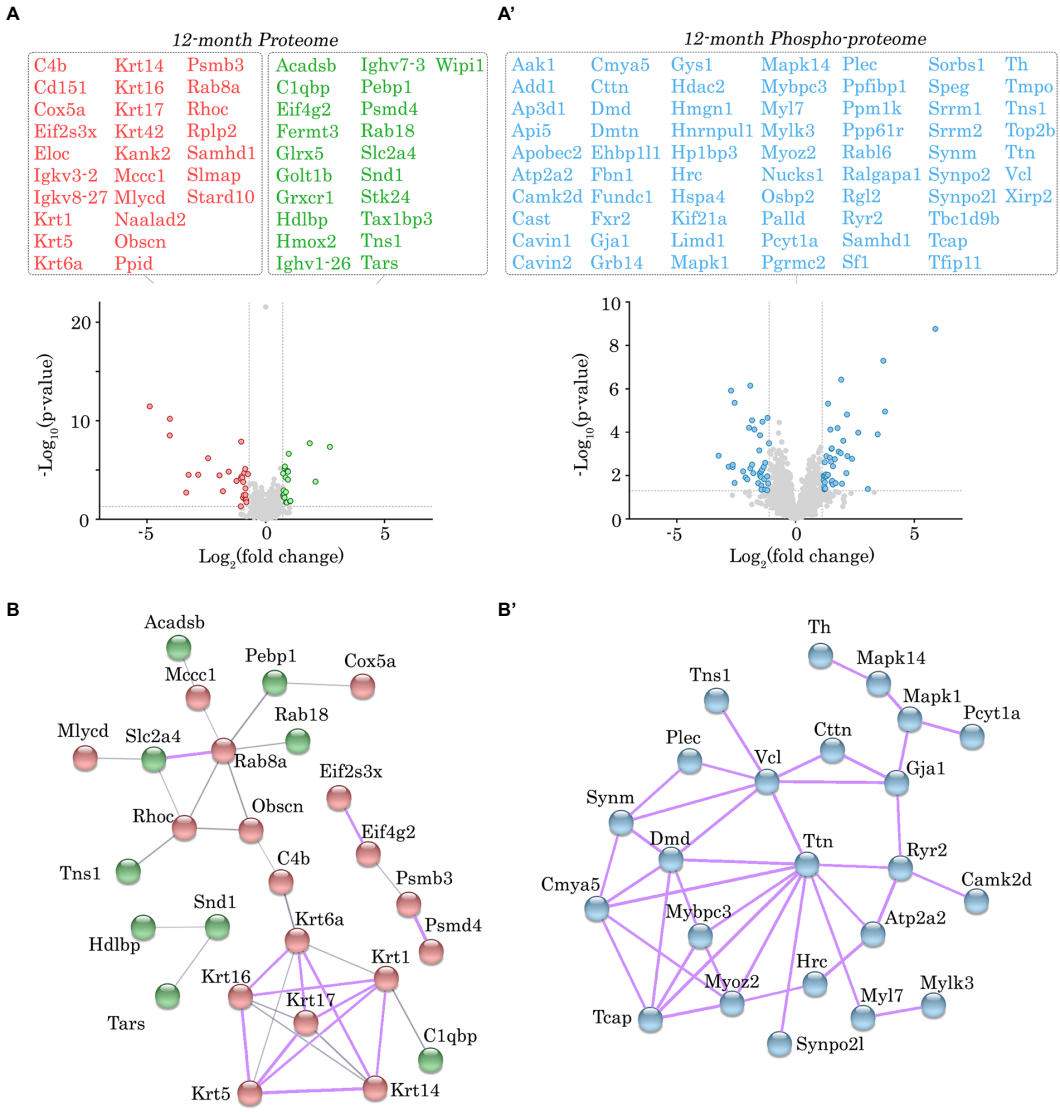
Proteomic and phospho-proteomic analysis of *Obscn-ΔIg58/59* atria at 12-months. **(A,A’)** Volcano plots of significantly up-regulated (green) or down-regulated (red) proteins **(A)** and significantly altered phospho-peptides (blue; **A’**) in *Obscn-ΔIg58/59* atria at 12-months. A total of 48 proteins out of 1708 detected exhibited significantly altered expression **(A)**, whereas 78 phospho-peptides out of 2,656 detected were significantly altered **(A’)** in 12-month-old *Obscn-ΔIg58/59* atria compared to wild-type; *n* = 5 biological samples per genotype; grey dotted lines represent thresholds of *p* < 0.05 and SD > 2. **(B,B’)** The physical and functional associations of the deregulated proteins **(B)** and phospho-proteins **(B’)** in *Obscn-ΔIg58/59* atria at 12-months were plotted using the STRING database (v.11.0b). Line thickness corresponds to the strength of the association. Protein networks with a high confidence score (>0.7) are highlighted in purple.

**Figure 5:**
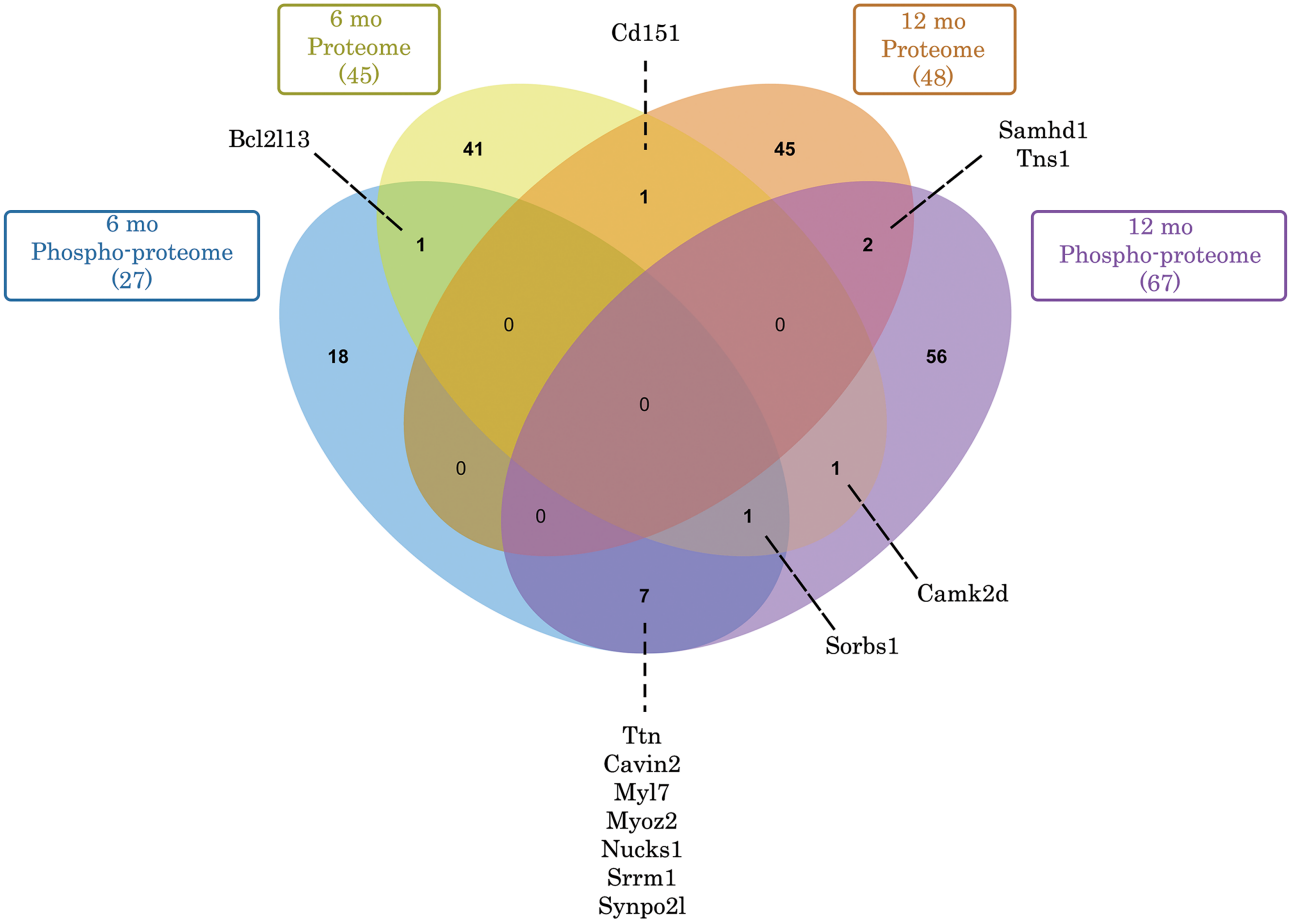
Venn diagram depicting commonly deregulated proteins and phospho-proteins in *Obscn-ΔIg58/59* atria through aging. Of the 45 (6-months) and 48 (12-months) proteins that exhibited significantly deregulated expression in *Obscn-ΔIg58/59* atria, only 1 (Cd151) was deregulated at both timepoints. In contrast, of the 27 (6-months) and 67 (12-months) deregulated phospho-proteins in *Obscn-ΔIg58/59* atria, a total of 8 (Ttn, Cavin2, Myl7, Myoz2, Nucks1, Srrm1, Synpo2l, and Sorbs1) were consistently deregulated through aging, albeit at different sites and/or affected in opposite directions at 6- versus 12-months (Supplementary Tables S2, S4). Lastly, CaMKIIδ exhibited deregulated expression at 6-months and altered phosphorylation at 12-months in *Obscn-ΔIg58/59* atria. Venn diagram generated with jvenn (84).

Given the lack of significant alterations in the levels of obscurin, the binding partners of obscurin-Ig58/59, and canonical Ca^2+^ cycling regulators, we performed proteomic and phospho-proteomic experiments using 6- and 12-month-old male wild-type and *Obscn-ΔIg58/59* atrial tissue (*n* = 5 hearts per group) to obtain a more comprehensive molecular profile of the *Obscn-ΔIg58/59* atria. At 6-months, we identified 45 proteins (out of 1700 detected) that exhibited significantly altered expression levels (Figure 3A; Supplementary Table S1; Supplementary Data Sheet 1) and 30 phospho-peptides (out of 1,401 detected) originating from 27 different proteins that displayed altered phosphorylation levels (Figure 3A’; Supplementary Table S2; Supplementary Data Sheet 2). By 12-months, we identified 48 proteins (out of 1708 detected) with altered expression levels (Figure 4A; Supplementary Table S3; Supplementary Data Sheet 3) and 78 phospho-peptides (out of 2,656 detected) corresponding to 67 different proteins that exhibited altered phosphorylation levels (Figure 4A’; Supplementary Table S4; Supplementary Data Sheet 4). Of the 45–48 affected proteins and 27–67 affected phospho-proteins in *Obscn-ΔIg58/59* atria, relatively few were commonly deregulated throughout aging (Figure 5).

In order to discover the functional relationships and/or physical associations shared by the deregulated proteins or phospho-proteins in *Obscn-ΔIg58/59* atria through aging, we performed a network analysis using the publicly available STRING database (v.11.0b) (22). At 6-months, there was a single network exhibiting a high confidence interaction score (>0.7) among the proteins displaying deregulated expression in *Obscn-ΔIg58/59* atria (Figure 3B), which consisted of ubiquitin-specific protease 14 (*Usp14*) and the proteasome subunit alpha type-2 (*Psma2*), possibly reflecting deregulated protein degradation pathways. Also connected to this network, albeit with a medium confidence interaction score (>0.4), is the purine biosynthetic enzyme, phosphoribosylaminoimidizole carboxylase/succinocarboxamide synthetase (*Paics*), possibly implicating altered DNA synthesis, intracellular signaling, and/or metabolic processes (31). On the other hand, STRING analysis of the deregulated phospho-proteins at 6-months revealed a high confidence network (Figure 3B’) comprised of sarcomeric proteins (i.e., titin, *Ttn*; myosin light chain 7, *Myl7*) and cytoskeletal proteins localizing to the Z-disc (i.e., myozenin, *Myoz2*; synaptopodin 2-like, *Synpo2l*; LIM domain binding protein 3, *Ldb3*). This network also included β-taxilin, a muscle-specific member of the taxilin family of vesicular trafficking regulators that is proposed to regulate myoblast differentiation (32). At 12-months, we observed a significant clustering of intermediate filament proteins (i.e., keratins, *Krt1, Krt5, Krt6a, Krt14, Krt16, Krt17*), in addition to a smaller high confidence network of protein homeostasis regulators (i.e., eukaryotic translation initiation factor 2, *Eif2s3x*; eukaryotic translation initiation factor 4 gamma 2, *Eif4g2*; proteasome 20s subunit beta 3, *Psmb3*; 26S proteasome regulatory subunit 4, *Psmd4*) that all exhibited reduced expression levels in *Obscn-ΔIg58/59* atria (Figure 4B). Lastly, STRING analysis of the deregulated phospho-proteins at 12-months revealed an extensive high confidence network comprised of 23 interconnected sarcomeric proteins, cytoskeletal proteins, ion channels, Ca^2+^ regulators, and kinases (Figure 4B’). Of note, titin represents the most prominent node in the phospho-proteomic network at both timepoints, forming functional and/or physical associations with 4/6 (66%) and 10/23 (43%) of the phospho-proteins that were deregulated at 6- or 12-months, respectively. Given that titin is a binding partner of the obscurin Ig58/59 module, this suggests that the disruption of the obscurin/titin complex could be integral to the deregulated phosphorylation events in *Obscn-ΔIg58/59* atria.

**Figure 6:**
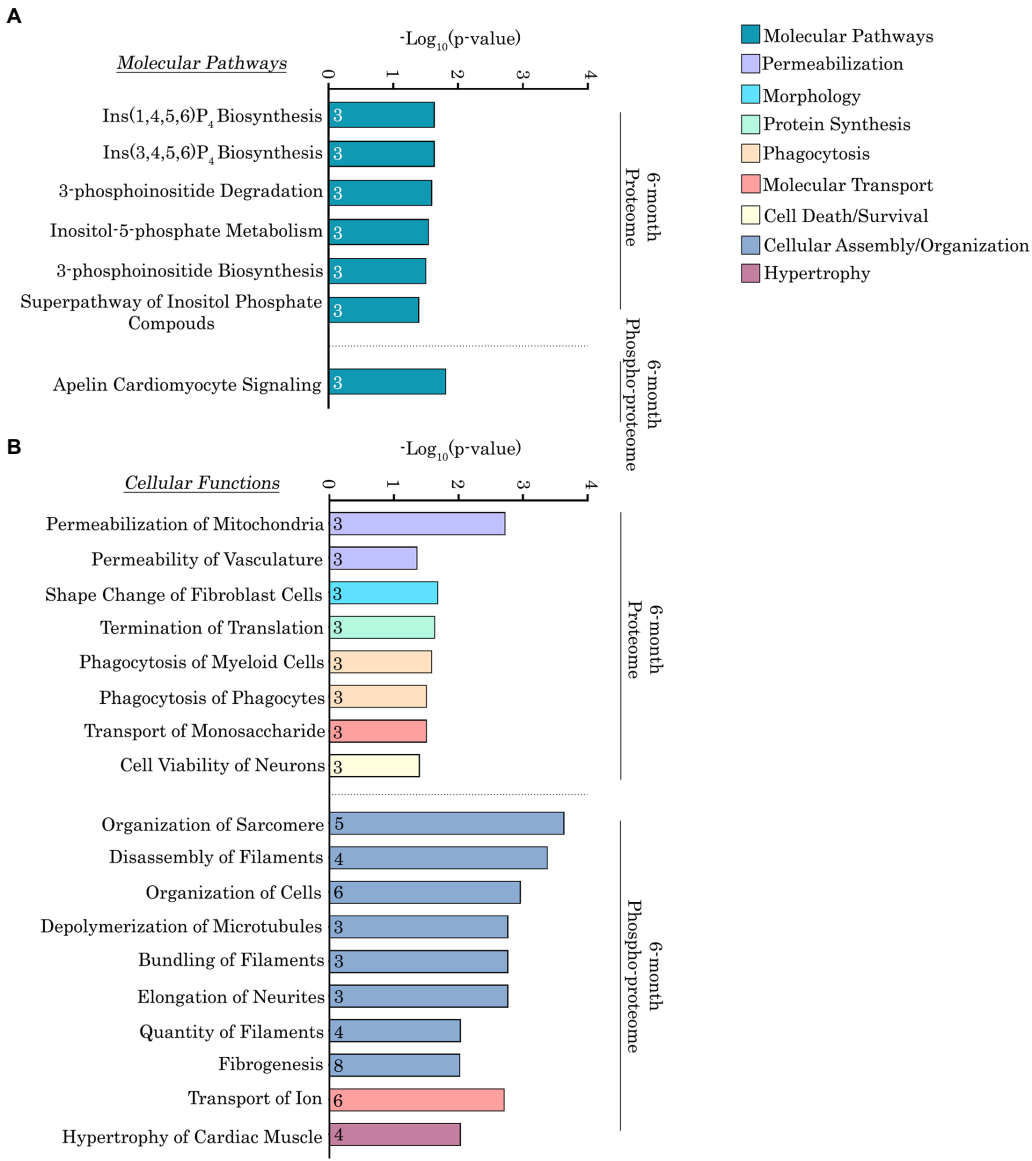
Ingenuity pathway analysis of the deregulated proteins and phospho-proteins in *Obscn-ΔIg58/59* atria at 6-months. **(A,B)** The molecular pathways **(A)** and cellular functions **(B)** associated with proteins that exhibited significantly altered expression or phosphorylation in 6-month-old *Obscn-ΔIg58/59* atria. The number of proteins associated with each biological process is indicated within the respective bar.

**Figure 7:**
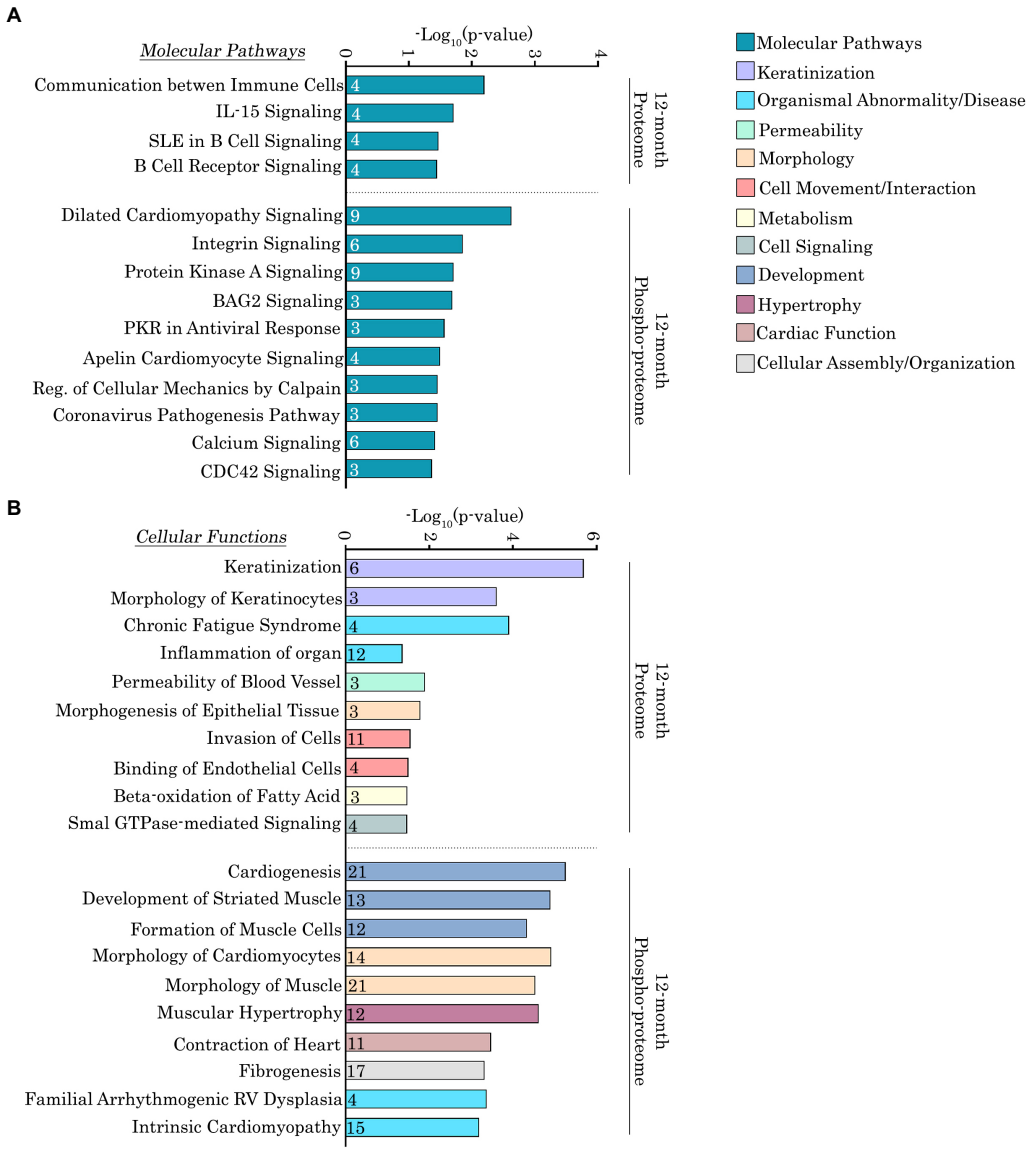
Ingenuity pathway analysis of the deregulated proteins and phospho-proteins in *Obscn-ΔIg58/59* atria at 12-months. **(A,B)** The molecular pathways **(A)** and cellular functions **(B)** associated with proteins that exhibited significantly altered expression or phosphorylation in 12-month-old *Obscn-ΔIg58/59* atria. The number of proteins associated with each biological process is indicated within the respective bar.

To more quantitatively delineate the major molecular pathways and cellular functions that were impacted by the Ig58/59 deletion, we performed an enrichment analysis on the proteins that exhibited significantly altered expression or phosphorylation levels (Figures 6A,B, 7A,B; Supplementary Tables S5–S8). At 6-months, proteins exhibiting altered expression were associated with the regulation of inositol phosphate metabolism (Figures 6A,B; Supplementary Table S5), whereas at 12-months, proteins exhibiting altered expression largely belonged to the keratin subfamily of intermediate filaments (Figures 7A,B; Supplementary Table S7). Additionally, proteins displaying altered phosphorylation were primarily associated with the regulation of cellular assembly/organization (i.e., organization of sarcomeres, filaments, and microtubules), ion transport, and cardiac hypertrophy at 6-months (Figures 6A,B; Supplementary Table S6) and various signaling cascades (i.e., protein kinase A; PKA, integrin, apelin, and Ca^2+^ cycling), striated muscle development, formation, morphology, and hypertrophy, as well as cardiomyopathy and familial arrhythmogenic right ventricular dysplasia at 12-months (Figures 7A,B; Supplementary Table S8).

Given the large number of deregulated proteins and phosphorylation events identified in our proteomic screen, we decided to focus on (1) direct alterations to obscurin, and (2) proteins that belong to enriched molecular pathways or cellular functions *and* represent nodes within high confidence protein networks identified *via* STRING analysis. Of note, we discuss these deregulated proteins and phospho-proteins in terms of their canonical protein class since many of them belong to multiple affected cellular processes and/or protein interaction networks.

### Alterations in obscurin in aging *Obscn-ΔIg58/59* atria

At 12-months of age, we observed a significant, yet modest, reduction (~1.8 fold) in the expression levels of obscurin in *Obscn-ΔIg58/59* atria compared to wild-type (Figure 4A; Supplementary Table S3). Of note, the lack of statistical significance in our immunoblotting analysis (Figures 1C,C’) that revealed only a trend toward decreased obscurin expression in *Obscn-ΔIg58/59* atria (~1.1 fold; *p* = 0.3), is most likely due to the reduced sensitivity of the immunoblotting technique compared to proteomics. Nevertheless, complete knockout or down-regulation of obscurin in striated muscles has been linked to major structural defects, including the disorganization of the longitudinal SR (33), disrupted thick filament assembly (34) and lateral alignment of myofibrils (35), loss of dystrophin at costameres, and alterations in the arrangement of the subsarcolemmal microtubule lattice (36). Therefore, this moderate reduction in obscurin expression in aged *Obscn-ΔIg58/59* atria could indicate a mild loss in the structural integrity of the myofibril, the cytoskeleton, and/or the SR membranes. Accordingly, obscurin expression levels are also reduced in human cardiac biopsies carrying DCM-linked point mutations in *OBSCN* (E963K, V2161D, or D5966N) (37), substantiating that obscurin haploinsufficiency is pathogenic in the heart.

### Alterations in cytoskeletal and structural regulators in aging *Obscn-ΔIg58/59* atria

In addition to the essential cytoskeletal protein obscurin, a striking portion of the deregulated proteins and phospho-proteins in aging *Obscn-ΔIg58/59* atria were canonical cytoskeletal and structural regulators. In particular, the Ig58/59 deletion induced profound abnormalities in the expression and/or phosphorylation of intermediate filaments, sarcomeric proteins, Z-disk-associated and/or actin-linked cytoskeletal modulators, and structural components of the costamere.

#### Intermediate filaments: Keratins

At 12-months, the expression levels of seven different keratin isoforms, including keratins 1, 5, 6A, 14, 16, 17, and 42, were significantly reduced in *Obscn-ΔIg58/59* atria (Figure 4A; Supplementary Table S3). Although keratins have not been extensively studied in the heart, studies evaluating keratin 19-deficient skeletal muscles show that they contribute to the organization of the costamere and the development of contractile force. Therefore, these findings indicate a drastic loss of intermediate filament proteins that are integral to the organization of costameres and force development in striated muscles (38, 39).

#### Sarcomeric cytoskeleton: Titin, T-cap, MyBPC-3, and Myosin Light Chain 7

Our phospho-proteomic analysis revealed age-related alterations in the phosphorylation status of titin in *Obscn-ΔIg58/59* atria. In particular, we observed decreased levels of a bi-phosphorylated peptide, Ser34063/Ser-Thr-Tyr_34062-34,080_, localizing to the titin M-band interdomain sequence 2 (Mis2) in *Obscn-ΔIg58/59* atria compared to wild-type at 6-months (Figure 3A’; Supplementary Table S2). The physiological function(s) of these phosphorylation events are not yet known, however the titin Mis2 region has been previously established as a binding site for DRAL/FHL2 (40). DRAL/FHL2 is a member of the four and a half LIM domain protein family that is thought to target metabolic enzymes to the M-band *via* binding to titin Mis2 (2, 40). Therefore, the deregulated phosphorylation of titin within Mis2 in *Obscn-ΔIg58/59* atria could potentially influence metabolic complexes that localize to the M-band. Along these lines, recent proteomic studies from our group and others conducted in heart or skeletal muscle where obscurin is either mutated (29) or deleted (41) have also reported alterations in proteins involved in metabolism, specifically lipid catabolism and amino acid metabolism (29) or glycogen metabolism (41).

Titin phosphorylation was also deregulated in *Obscn-ΔIg58/59* atria at 12-months at three distinct locations: a bi-phosphorylated peptide corresponding to the Mis2 region of titin (Ser33875/Ser33880), a site within the M-band (Ser34470), and a site within titin-Ig76/77 (Ser9459; Figure 4A’; Supplementary Table S4). Interestingly, the Ser34470 residue which exhibited enhanced phosphorylation resides within the third Lys-Ser-Pro (KSP) motif in titin-Mis4. Of note, the KSP motifs have been shown to be highly phosphorylated during development in muscle and are thought to regulate the assembly of the M-band (42). Thus, these findings are in line with the significantly increased atrial mass observed in *Obscn-ΔIg58/59* atria at 12-months (10) in addition to our pathway analysis that indicated alterations in developmental processes such as cardiogenesis, striated muscle development, and the morphology of cardiomyocytes.

Telethonin, also known as Titin-cap or T-cap, is a Z-disk associated protein that binds to titin’s extreme NH_2_-terminal Ig1/2 domains where it is proposed to regulate sarcomeric development, stability, and stretch responses (43, 44). Our proteomic screen revealed reduced phosphorylation of T-cap at Ser161 in *Obscn-ΔIg58/59* atria at 12-months (Figure 4A’; Supplementary Table S4). Candasamy et al. (44) previously reported that endogenous T-cap is constitutively bi-phosphorylated by both protein kinase D and CaMKII at Ser157 and Ser161 in rodent myocardia, and that disruption of these phosphorylation events results in disorganized t-tubule structures and abnormal Ca^2+^ cycling. Given that the COOH-terminal region of T-cap containing Ser157/Ser161 binds accessory proteins that localize to t-tubules, it has been further suggested that Ser157/Ser161 phosphorylation may regulate T-cap’s ability to serve as an “adapter protein” linking t-tubules to the Z-disk (44). An important observation is that the obscurin-Ig58/59 binding site on titin (Ig9/10) exists in relative proximity to T-cap’s binding site to titin (i.e., titin-Ig1/2). It is therefore conceivable that the Ig58/59 deletion could possibly disrupt the titin/T-cap complex at the Z-disk and/or influence the nearby regulatory networks that mediate Ser161 phosphorylation. Along these lines, the observed reduction of Ser161 phosphorylation in 12-month *Obscn-ΔIg58/59* atria could possibly lead to disorganized t-tubule morphology, disrupted Ca^2+^-induced Ca^2+^-release, and potentially contribute to the development of arrhythmia.

Lastly, we saw altered phosphorylation levels of proteins localizing to the thick filament in *Obscn-ΔIg58/59* atria through aging, including myosin light chain 7 and myosin binding protein-C (MyBP-C). In particular, we observed a ~ 60-fold decrease in the phosphorylation of Ser22 on myosin light chain 7 at 6-months (Figure 3A’; Supplementary Table S2), which is the atrial myosin regulatory light chain isoform. The exact function of this phosphorylation event is not thoroughly characterized, though it was previously shown to be mediated by myosin light chain kinase in response to α-adrenergic signaling (45). Moreover, phosphorylation of Ser23 on myosin light chain 7, immediately adjacent to the phosphorylation site Ser22 that was deregulated in *Obscn-ΔIg58/59* atria at 6-months, was significantly reduced at 12-months (Figure 4A’; Supplementary Table S4). Furthermore, we also identified a novel phosphorylation event on cardiac MyBP-C, Ser268, which exhibited a ~ 2.5-fold reduction in phosphorylation in 12-month old *Obscn-ΔIg58/59* atria (Figure 4A’; Supplementary Table S4). Importantly, this phosphorylation site localizes within the M-motif (between Ig domains C1 and C2), which is well established as a phosphorylation ‘hot-spot’ for cardiac MyBP-C. Previous studies have shown that reduced phosphorylation of cardiac MyBP-C within the M-motif is associated with the development of heart failure in mice and results in reduced rates of contraction and relaxation. It is therefore possible that reduced phosphorylation of Ser268 on cardiac MyBP-C and/or Ser23 on myosin light chain 7 could indicate deregulation of contractility in *Obscn-ΔIg58/59* atria at 12-months.

#### Actin-linked and/or Z-disk-associated cytoskeleton: Plectin, Cortactin, Myozenin, Synaptopodin 2-l, LIM-domain-binding protein 3, Myospryn

Plectin, a cytoskeletal protein that forms physical links between actin, microtubules, and intermediate filaments, exhibited increased phosphorylation at Ser4415 in *Obscn-ΔIg58/59* atria at 12-months (Figure 4A’; Supplementary Table S4). Additionally, cortactin, a scaffold protein that regulates the polymerization and stabilization of the actin cytoskeleton, exhibited decreased phosphorylation at a tri-phosphorylated peptide, Thr401/Ser405/Ser407 (Figure 4A’; Supplementary Table S4). Phosphorylation of the Thr401/Ser405 residues on cortactin, mediated by Erk (Ser405) (46), Akt (Thr401/Ser405) (47), and/or PKCδ (Ser405) (48), promotes actin polymerization and cell migration *via* enhancing the interaction between cortactin and actin nucleation promoting factors. Together, these results demonstrate alterations in the phosphorylation status of proteins regulating the assembly and organization of the actin cytoskeleton in 12-month *Obscn-ΔIg58/59* atria.

Our proteomic results indicated deregulated phosphorylation of three additional actin-associated proteins in *Obscn-ΔIg58/59* atria. In particular, phosphorylation of synaptopodin 2-like, which is a member of the synaptopodin family of proteins that regulate actin polymerization at the Z-disk, was altered at Ser89/Ser-Thr_83-126_ and Thr88/Ser97 at 6-months and 12-months, respectively (Figures 3A’, 4A’; Supplementary Tables S2, S4). Of note, Thr88 represents a novel phosphorylation event on synaptopodin 2-like. In addition, LIM-domain-binding protein 3, also known as Z-band alternatively spliced PDZ motif protein (ZASP), displayed decreased phosphorylation at Thr119/Ser123 in *Obscn-ΔIg58/59* atria at 6-months (Figure 3A’; Supplementary Table S2). LIM-domain-binding protein 3 (ZASP) is a cytoskeletal protein that regulates Z-disk integrity and signal transduction through forming complexes with an array of Z-disk proteins, including α-actinin-2 (49, 50), myozenin (51, 52), myotilins (myotilin, myopalladin, and palladin) (52), and telethonin/Tcap (53). Accordingly, the phosphorylation status of ZASP-interactive myozenin was also disrupted at phosphorylated peptides Ser95/Ser116 and Thr111/Ser116/Ser-Thr-Tyr_92-132_ at 6-months, and Thr107 at 12-months (Figures 3A’, 4A’; Supplementary Tables S2, S4). Together, these findings provide strong evidence that major cytoskeletal signaling complexes at the level of the Z-disk/thin filament are disrupted in *Obscn-ΔIg58/59* atria throughout aging.

#### Dystrophin complex at the costamere: Dystrophin, Vinculin, Synemin

Several proteins that serve as integral components of the dystrophin/dystroglycan complex and/or form structural links between the costamere and the cytoskeleton exhibited altered phosphorylation in *Obscn-ΔIg58/59* atria at 12-months. In particular, synemin, an intermediate filament protein that mediates the lateral transmission of force and maintains the structural integrity of the myofibril during mechanical stress, exhibited increased phosphorylation at Ser1087 in *Obscn-ΔIg58/59* atria (Figure 4A’; Supplementary Table S4). Synemin is present at the level of the Z-disk where it interacts with α-actinin, desmin, vinculin, and components of the dystrophin glycoprotein complex (54). Interestingly, vinculin, which links integrins to the actin cytoskeleton, exhibited increased phosphorylation at Ser721 in *Obscn-ΔIg58/59* atria at 12-months (Figure 4A’; Supplementary Table S4). Furthermore, the abundance of a phospho-peptide corresponding to dystrophin, Ser-Thr_3624-3,664_, was decreased (Figure 4A’; Supplementary Table S4). Together, these results suggest phosphorylation defects in protein complexes that contribute to the formation of cytoskeletal links between the sarcomere and the extracellular matrix.

#### Gap junctions: Connexin-43

We also identified altered phosphorylation levels of connexin-43, the core protein that comprises gap junctions, in *Obscn-ΔIg58/59* atria at 12-months. In particular, we saw increased phosphorylation of connexin-43 at Ser325/Thr326, and decreased levels of the phospho-peptide, Ser-Thr_320-345_ (Figure 4A’; Supplementary Table S4). Notably, the phosphorylation of Ser325 on connexin-43, along with nearby residues Ser328 and Ser330, is mediated by casein kinase 1 and has been shown to stabilize the formation of gap junctions at the intercalated disc (55). Thus, up-regulation of pSer325 on connexin-43 could indicate enhanced gap junction formation in *Obscn-ΔIg58/59* atria at 12-months and potentially impacting synchronous cardiomyocyte contraction therefore underlying arrhythmic events.

In summary, our proteomic screen revealed extensive abnormalities in the expression and phosphorylation status of major structural regulators in aging *Obscn-ΔIg58/59* atria, including proteins that regulate the assembly and organization of the myofibril, form gap junctions, physically link the sarcomere to the surrounding membranes, and are integral components of the Z-disk associated cytoskeleton. Notably, obscurin and titin, two key cytoskeletal regulators, interact at the level of the Z-disk (8). Given that the Z-disk is a region that integrates proteins of the sarcomere and the surrounding cellular structures, including the cytoskeleton, intercalated disc, and plasma membrane (56), it is interesting to speculate that the disrupted binding between obscurin-Ig58/59 and titin could severely affect the stability and/or regulation of protein complexes that localize to this region. Moreover, many cytoskeletal proteins that localize to the Z-disk, particularly those forming connections to the extracellular matrix *via* costameres, aid in the transmission of force and mediate mechanical transduction pathways (56). Therefore, our proteomics and phospho-proteomics findings could reflect pathological alterations in the stabilization of the myofibril during mechanical stress in *Obscn-ΔIg58/59* atria.

### Alterations in regulatory proteins and signaling mediators in aging *Obscn-ΔIg58/59* atria

Our proteomic and phospho-proteomic analysis also revealed alterations in the expression and phosphorylation of regulatory proteins and signaling mediators in aging *Obscn-ΔIg58/59* atria. Specifically, we observed alterations in canonical regulators of Ca^2+^ cycling and major protein kinases that could potentially contribute to the development of atrial fibrillation in *Obscn-ΔIg58/59* mice.

#### Calcium cycling proteins: SERCA2, SERCA3, RyR2, HRC

Our phospho-proteomic screen and subsequent enrichment analysis identified several differentially phosphorylated Ca^2+^ cycling regulators in *Obscn-ΔIg58/59* atria at 6- and 12-months. At 6-months, we identified a novel phosphorylation site localized within the hydrolase domain of SERCA3, Ser729, which was significantly reduced in *Obscn-ΔIg58/59* atria (Figure 3A’; Supplementary Table S2). SERCA3 was originally thought to be exclusively expressed in non-muscle tissues, however, SERCA3 isoforms were ultimately detected in normal human LV tissue as well (57). Notably, a significant distinction between SERCA3 and the more abundantly expressed SERCA2a isoform is that SERCA3 is unable to bind PLN (58). Given that loss of binding between Ig58/59 and PLN in *Obscn-ΔIg58/59* atria could lead to enhanced inhibition of SERCA2 (*via* loss of Ig58/59-mediated sequestration of PLN), it is possible that the decreased phosphorylation of SERCA3 at Ser729 at 6-months could serve as a compensatory response. Moreover, at 12-months, we observed a significant reduction in the levels of the SERCA2 phospho-peptide, Ser-Thr-Tyr_372-397_, demonstrating potential abnormalities in Ca^2+^ reuptake into the SR through aging (Figure 4A’; Supplementary Table S4).

Histidine rich Ca^2+^ binding protein (HRC), a protein that localizes to the SR lumen where it regulates Ca^2+^ storage and release (59), exhibited reduced phosphorylation at Ser272 in *Obscn-ΔIg58/59* atria at 12-months (Figure 4A’; Supplementary Table S4). Although this phosphorylation site has not been experimentally characterized, previous studies have suggested that phosphorylation of HRC by casein kinase II regulates RyR2 function in skeletal muscle (59, 60). Importantly, we also observed up-regulation of phosphorylated Ser2810 (Ser2811 in humans) on RyR2 in *Obscn-ΔIg58/59* atria at 12-months (Figure 4A’; Supplementary Table S4). Hyper-phosphorylation of RyR2, specifically at the canonical Ser2808 and Ser2814 sites (human notation), has been strongly linked to enhanced RyR2 open probability and susceptibility to arrhythmia (25, 26). Although there is still controversy regarding the roles of individual RyR2 phosphorylation sites and their potential functional redundancies, it is generally accepted that the “phosphorylation hot-spot” in RyR2 encompassing human Ser2808 through Ser2814 (26) is an effective modulator of Ca^2+^ release from the SR. In addition to the Ser2808 and Ser2814 sites which are regulated by CaMKII and/or PKA, there are two additional sites within the hot-spot, Thr2810 and Ser2811, for which less information is known, although both are predicted to impact RyR2 function similarly to Ser2808 and Ser2814 (26, 27). Therefore, hyper-phosphorylation of RyR2 at Ser2810 (human Ser2811) combined with altered HRC phosphorylation in *Obscn-ΔIg58/59* atria could potentially lead to abnormal Ca^2+^ release and/or Ca^2+^ leak from the SR in *Obscn-ΔIg58/59* atria at 12-months.

#### Kinases: CaMKIIδ, SPEG, MAPKs, myosin light chain kinase 3

In addition to proteins directly regulating Ca^2+^ homeostasis, we also identified alterations in the expression and phosphorylation of several kinases in our proteomic screen including Ca^2+^/calmodulin-dependent protein kinase δ (CaMKIIδ) and striated muscle preferentially expressed gene (SPEG). CaMKIIδ is one of the major protein kinases that regulates Ca^2+^ dynamics in the heart *via* phosphorylation of Ca^2+^ handling proteins in response to physiological and/or pathological stimuli (61, 62). Importantly, the expression level of CaMKIIδ was ~2.5 fold lower in *Obscn-ΔIg58/59* atria compared to wild-type at 6-months (Figure 3A; Supplementary Table S1). At 12-months, CaMKIIδ abundance was no longer altered, but its phosphorylation was increased at both Thr331 and another site within the Ser-Thr_323-344_ region (Figure 4A’; Supplementary Table S4). The physiological significance of pThr331 has not yet been experimentally determined. However, the deregulation of CaMKIIδ in aged *Obscn-ΔIg58/59* male atria along with its preeminent role in cardiac hypertrophy makes it a key target for future investigation.

Our phospho-proteomic analysis also identified a novel phosphorylation site, Ser2200, that localizes to the inter-kinase region of SPEG, a paralog of obscurin that arose from gene duplication of *OBSCN*, that was significantly decreased in *Obscn-ΔIg58/59* atria at 12-months (Figure 4A’; Supplementary Table S4). SPEG, sharing high homology to obscurin, also possesses two tandem kinase domains at its COOH-terminus (termed SK1 and SK2, highly homologous to obscurin Kin1 and Kin2) that have been implicated in the regulation of Ca^2+^ homeostasis (63). In addition to Ser2200, SPEG also exhibited decreased phosphorylation at Ser2182, which is located within the same inter-kinase region (Figure 4A’; Supplementary Table S4). The functions of these phosphorylation events are not known, but they could potentially affect the substrate specificities and/or activities of SPEG SK1, which phosphorylates junctophilin 2, and/or SPEG SK2, that phosphorylates SERCA2 and possibly RyR2 (63).

Additionally, phosphorylation of myosin light chain kinase 3 was decreased at Ser155 in *Obscn-ΔIg58/59* atria at 12-months (Figure 4A’; Supplementary Table S4). Although the function of this site is not precisely known, this finding could corroborate the reduction in phosphorylation of its substrate, myosin regulatory light chain 7, that we also observed in *Obscn-ΔIg58/59* atria (Figure 4A’; Supplementary Table S4). Lastly, we observed altered phosphorylation levels of mitogen-activated protein kinase 1 (MAPK1) and 14 (MAPK14) at Thr183/Thr188 (up-regulated) and Thr185 (down-regulated), respectively (Figure 4A’; Supplementary Table S4). MAPKs are a family of highly conserved signaling mediators that regulate a diverse set of cellular processes such as proliferation, cell death/survival, transcription, migration, and differentiation by phosphorylating hundreds of downstream targets. In the heart, MAPK1 and MAPK14 isoforms regulate cardiac development and differentiation and promote the hypertrophic response (64). Importantly, Thr183/Thr188 and Thr185 reside within the regulatory loop of MAPK1 and MAPK14. In fact, Thr183 in MAPK1 is part of the canonical Thr-Glu-Tyr motif, which is phosphorylated by the upstream kinase, MEK1/2 (65, 66).

Lastly, tyrosine hydroxylase, the rate limiting enzyme involved in the synthesis of catecholamines such as epinephrine and norepinephrine, exhibited reduced phosphorylation at Thr30 in *Obscn-ΔIg58/59* atria at 12-months (Figure 4A’; Supplementary Table S4). While not a kinase itself, alteration of tyrosine hydroxylase could impact adrenergic activity and/or downstream PKA signaling in *Obscn-ΔIg58/59* atria.

## Conclusion

Collectively, our proteomics and phospho-proteomics data demonstrated extensive alterations in the expression and phosphorylation status of proteins involved in diverse cellular processes, including major Ca^2+^ cycling regulators, protein kinases, and cytoskeletal protein complexes associated with the Z-disk that likely drive atrial structural remodeling and arrhythmogenesis in *Obscn-ΔIg58/59* male mice. It is interesting to note the lack of proteins that consistently exhibit altered expression in *Obscn-ΔIg58/59* atria at both timepoints (Figure 5). In contrast, multiple phospho-proteins are affected throughout aging in *Obscn-ΔIg58/59* atria including titin (*Ttn*), caveolae associated protein 2 (*Cavin2*), myosin light chain 7 (*Myl7*), myozenin 2 (*Myoz2*), nuclear casein kinase and cyclin-dependent kinase substrate 1 (*Nucks1*), sorbin and SH3 domain containing 1 (*Sorbs1*), serine/arginine repetitive matrix 1 (*Srrm1*), and synaptopodin 2-like (*Synpo2l*; Figure 5). Notably, several of these proteins (i.e., titin, myosin light chain 7, myozenin, and synaptopodin 2-like) also constitute core components of the phospho-proteomic STRING networks at both timepoints, comprising 4/6 of the functionally and/or physically interconnected phospho-proteins at 6-months or contribute to a much larger network composed of 23 interconnected phospho-proteins at 12-months. Together, these observations suggest that these commonly deregulated phospho-proteins could represent key players in disease development due to deletion of obscurin-Ig58/59, and that disrupted phosphorylation events could largely contribute to the progressive pathologies that manifest through aging.

**Figure 8:**
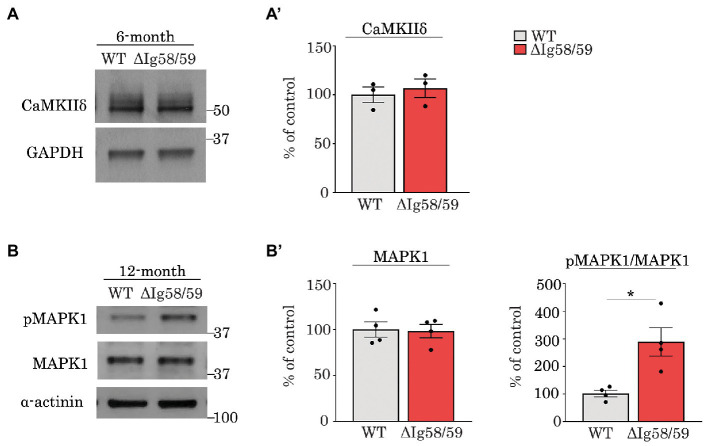
Immunoblot analysis confirmed up-regulation of phosphorylated MAPK1 in 12-month old *Obscn-ΔIg58/59* atria. **(A,A’)** Representative immunoblots **(A)** and relative quantifications **(A’)** did not reveal statistically significant alterations in the expression of CaMKIIδ in lysates prepared from 6-month-old wild-type and *Obscn-ΔIg5859* atria; *t*-test, *p* = 0.62. **(B,B’)** Representative immunoblots **(B)** and relative quantifications **(B’)** revealed an up-regulation of phosphorylated MAPK1 (also known as ERK2) at its canonical activation motif, pThr183/Tyr185, in *Obscn-ΔIg5859* atria at 12-months but no differences in total MAPK1/ERK2 levels; *t*-test, *p < 0.05, α-actinin and GAPDH served as loading controls; *n* = 3–4 animals per group; data points represent the average of at least two technical replicas; quantification of phosphorylation levels are normalized to total MAPK1/ERK2 levels.

Along these lines, our proteomics analysis revealed alterations in the expression and/or phosphorylation status of major protein kinases in aging *Obscn-ΔIg58/59* atria, including CaMKIIδ and MAPK1 and 14. To corroborate our proteomics findings and underscore the contribution of deregulated phosphorylation to the *Obscn-ΔIg58/59* disease phenotype, we performed immunoblots evaluating the expression and phosphorylation levels of two major deregulated kinases in *Obscn-ΔIg58/59* atria that are amenable to validation due to the availability of relevant (phospho)-antibodies. In contrast to our proteomics results (Figure 3A; Supplementary Table S1), we were not able to confirm decreased expression of CaMKIIδ in *Obscn-ΔIg58/59* atria at 6-months *via* immunoblot analysis (Figures 8A,A’), possibly reflecting the reduced sensitivity of immunoblotting techniques compared to proteomics, or a potential artifact in our proteomics data. We next evaluated the phosphorylation levels of MAPK1 (also known as ERK2) at pThr183/pTyr185, given that our phospho-proteomic screen revealed a 2.35-fold increase in the levels of the bi-phosphorylated peptide, Thr183/Thr188, in *Obscn-ΔIg58/59* atria at 12-months (Figure 4A’; Supplementary Table S4). Consistent with our proteomics results, our immunoblotting analysis revealed a 2.89-fold increase in the phosphorylation of the canonical activation motif, pThr183/pTyr185 (Figures 8B,B’), demonstrating up-regulated MAPK1 activity in 12-month *Obscn-ΔIg58/59* atria. Given the established role of MAPK1 in the hypertrophic response and atrial fibrillation (64, 67–69), this may contribute to the development of progressive remodeling and/or arrhythmogenesis in aging *Obscn-ΔIg58/59* atria.

In addition to potential hypertrophic remodeling mediated by MAPKs, the abundance of deregulated cytoskeletal proteins in *Obscn-ΔIg58/59* atria (particularly those localizing to the Z-disk) suggests abnormalities in the organization of sarcomeres and the cellular structures that form connections to the Z-disk (i.e., t-tubules, the intercalated disc, costameres). In particular, the dramatic reduction in keratin protein levels, and decreased phosphorylation of residues with defined (patho)physiological functions such as T-cap (Ser161), cortactin (Thr401/Ser405/Ser407), and connexin-43 (Ser325), implicate disrupted cytoskeletal structures, t-tubules, and intercalated discs in *Obscn-ΔIg58/59* atria at 12-months. Interestingly, we did not observe any major defects in myofibril or sarcomeric ultrastructure in our evaluation of *Obscn-ΔIg58/59* left ventricles (10), suggesting potential distinctions in the cellular and molecular pathogenesis of the Ig58/59 deletion between cardiac chambers. Along these lines, neither our immunoblotting nor our proteomics analysis revealed reduced phosphorylation of PLN (Thr17) or RyR2 (Ser2814) in aging *Obscn-ΔIg58/59* atria, which were both significantly decreased in *Obscn-ΔIg58/59* left ventricles (10). In contrast, RyR2 was hyperphosphorylated at a distinct site, Ser2810 (human Ser2811), in *Obscn-ΔIg58/59* atria at 12-months. Our future studies will more closely interrogate the cellular impacts of the Ig58/59 deletion specifically in atrial tissues to determine how chamber-specific molecular alterations caused by the Ig58/59 deletion affect atrial structure and function.

Our physiological evaluations of the *Obscn-ΔIg58/59* model revealed the presence of severe arrhythmia characterized by episodes of junctional escape and the sporadic loss of regular p-waves reminiscent of atrial fibrillation (10). Atrial fibrillation represents the most common type of sustained arrhythmia in humans and its prevalence increases substantially with aging (70, 71). The entire complex of structural, architectural, contractile, and electrophysiological alterations occurring in diseased atrial myocardium has recently established “atrial cardiomyopathy” as a new disease entity (72). Previous proteomic efforts aiming to characterize the molecular changes underlying atrial fibrillation and/or atrial cardiomyopathy in animals (73, 74) and humans (75–82) have similarly reported alterations in structural and metabolic proteins, ion channels and Ca^2+^ regulators (82). However comprehensive phospho-proteomic analyses remain scarce, although they are integral to deciphering the role of phosphorylation in the pathogenesis of atrial fibrillation (83). Nonetheless, the discovery of novel and/or uncharacterized phosphorylation events *via* phospho-proteomic screens must be further validated *in situ* and *in vivo* and investigated in terms of pathophysiological and functional relevance.

In summary, to our knowledge, the present study is the first to evaluate the atrial phospho-proteome through aging using a genetic model of spontaneous atrial arrhythmia and remodeling. Given the presence of both structural and regulatory proteins exhibiting deregulated expression and/or phosphorylation in aging *Obscn-ΔIg58/59* atria (including many phosphorylation events with unknown functions), our present findings reveal numerous molecular targets associated with novel and/or uncharacterized pathways to be interrogated in future studies and provides new mechanistic insights into atrial remodeling and dysfunction.

## Data availability statement

The data presented in the study are deposited in the Proteome Xchange Consortium via the Pride partner repository, accession number PXD028904.

## Ethics statement

The animal study was reviewed and approved by the Institutional Animal Care and Use Committee at the University of Maryland, School of Medicine. Written informed consent for participation was not obtained from the owners because the animal model that was used was generated in the Senior and Corresponding Author’s lab using a commercial source, therefore no written consent was required.

## Author contributions

AG: conceptualization, methodology, validation, formal analysis, investigation, writing original draft, and review and editing. WH: methodology, validation, formal analysis, review and editing. AB: methodology, formal analysis, review and editing. MK: methodology, validation, formal analysis, review and editing. AK-K: conceptualization, review and editing, supervision, project administration, and funding acquisition. All authors contributed to the article and approved the submitted version.

## Funding

This work was supported by the National Institutes of Health Training Program in Muscle Biology, T32 AR007592 to AG, and R01AR077106 to AK-K. Additional support was provided by the University of Maryland School of Pharmacy Mass Spectrometry Center (SOP1841-IQB2014).

## Conflict of interest

The authors declare that the research was conducted in the absence of any commercial or financial relationships that could be construed as a potential conflict of interest.

## Publisher’s note

All claims expressed in this article are solely those of the authors and do not necessarily represent those of their affiliated organizations, or those of the publisher, the editors and the reviewers. Any product that may be evaluated in this article, or claim that may be made by its manufacturer, is not guaranteed or endorsed by the publisher.
