# Supplementary material for: Alterations in cytoskeletal and Ca2+ cycling regulators in atria lacking the obscurin Ig58/59 module
Source: Front Cardiovasc Med. 2023 Apr 13;10:1085840. doi: 10.3389/fcvm.2023.1085840 (PMC10251194; doi:10.3389/fcvm.2023.1085840)
Supplement: Supplementary file 1 [file Table_1.pdf]

Supplemental Table 1. Proteins with significantly altered expression in *Obscn-ΔIg58/59* atria at 6-months.

Gene	Protein Name	Fold Change	P-value
ACP6	Acid phosphatase 6, lysophosphatidic	4.14	1.21E-02
ANXA6	Annexin A6	-11.83	2.05E-05
ATAD1	ATPase family, AAA domain containing 1	2.09	4.92E-02
BCL2L13	BCL2-like 13 (apoptosis facilitator)	-2.76	1.69E-02
BPGM	2,3-bisphosphoglycerate mutase	2.13	2.22E-03
CAMK2D	Calcium/calmodulin-dependent protein kinase II, delta	-2.49	2.60E-06
CAPRIN1	Cell cycle associated protein 1	3.36	2.54E-02
CAR3	Carbonic anhydrase 3	-3.02	1.95E-02
CD151	CD151 antigen	6.57	1.67E-02
CMAS	Cytidine monophospho-N-acetylneuraminic acid synthetase	2.75	1.09E-07
CORO1B	Coronin, actin binding protein 1B	2.21	1.76E-02
CSRP1	Cysteine and glycine-rich protein 1	-4.97	7.66E-03
DOCK10	Dedicator of cytokinesis 10	-3.61	4.67E-03
EIF4B	Eukaryotic translation initiation factor 4b	-2.88	2.16E-02
FTL1	Ferritin light polypeptide 1	-3.21	2.31E-07
GCSH	Glycine cleavage system protein H (aminomethyl carrier)	-8.11	4.78E-09
GLRX	Glutaredoxin	3.41	6.23E-03
GM12728	Peptidy-prolyl cis-trans isomerase A pseudogene 4_954.1	3.22	2.55E-02
HK2	Hexokinase 2	-3.80	5.11E-03
ITIH1	Inter-alpha trypsin inhibitor, heavy chain 1	2.23	2.52E-02
JUP	Junction plakoglobin	2.22	8.79E-05
KAT6B	K(lysine) acetyltransferase 6b	-3.76	2.41E-03
KTN1	Kinectin 1	-2.37	1.26E-02
LIMCH1	LIM and calponin homology domains 1	-9.22	4.24E-06
MFGE8	Milk fat globule EGF and factor V/VIII domain containing (lactadherin)	2.16	1.28E-02
MRPL10	Mitochondrial ribosomal protein L10	-2.08	2.73E-04
MRPS28	Mitochondrial ribosomal protein S28	3.00	7.80E-03
MSRB2	Methionine sulfoxide reductase B2	-2.90	1.10E-03
MTFR1L	Mitochondrial fission regulator 1-like	-2.86	3.89E-02
NDRG1	N-myc downstream regulated gene 1	2.44	1.45E-02
NEXN	Nexilin	18.99	5.59E-11
NHLRC2	NHL repeat containing 2	5.06	8.51E-05
NUDT4	Nudix (nucleoside diphosphate linked moiety X)-type motif 4	6.87	1.27E-03
NUMA1	Nuclear mitotic apparatus protein 1	-14.90	1.93E-03

PAICS	Phosphoribosylaminoimidazole carboxylase, phosphoribosylaminoribosylaminoimidazole, succinocarboxamide synthetase	2.18	2.95E-02
PDIA3	Protein disulfide isomerase associated 3	-2.38	6.11E-03
PSMA2	Proteasome subunit alpha 2	-7.00	1.10E-07
QARS	Glutaminyl-tRNA synthetase	4.41	4.35E-02
RANBP3	RAN binding protein 3	-3.88	1.01E-03
RPL34	Ribosomal protein L34	-68.72	2.94E-07
SFXN1	Sideroflexin 1	2.99	7.06E-03
SORBS1	Sorbin and SH3 domain containing 1	-3.93	4.50E-02
STX16	Syntaxin 16	-7.74	1.04E-04
UPF1	UPF1 regulator of nonsense transcripts homolog (yeast)	-58.14	9.47E-10
USP14	Ubiquitin specific peptidase 14	2.55	8.38E-07
