# Supplementary material for: Alterations in cytoskeletal and Ca2+ cycling regulators in atria lacking the obscurin Ig58/59 module
Source: Front Cardiovasc Med. 2023 Apr 13;10:1085840. doi: 10.3389/fcvm.2023.1085840 (PMC10251194; doi:10.3389/fcvm.2023.1085840)
Supplement: Supplementary file 2 [file Table_2.pdf]

Supplemental Table 2. Proteins with significantly altered phosphorylation in *Obscn-ΔIg58/59* atria at 6-months.

UniProt Accession	Gene	Protein Name	Site	Fold Change	P-value
E9Q1I5	*ABCC1*	ATP-binding cassette, sub-family C (CFTR/MRP), member 1	Ser289	-3.12	2.68E-02
E9Q559	*ATP2A3*	ATPase, Ca++ transporting, ubiquitous	Ser729*	-7.38	1.31E-02
P59017	*BCL2L1L13*	BCL2-like 13 (apoptosis facilitator)	Thr389	-3.87	1.75E-02
Q63918	*CAVIN2*	Caveolae associated 2	Ser/Thr$_{315-351}$	-3.81	1.28E-02
Q923F1	*CLNS1A*	Chloride channel, nucleotide-sensitive, 1A	Ser100	4.34	2.01E-02
Q76LL6	*FHOD3*	Formin homology 2 domain containing 3	Thr413; Ser417	-16.69	1.63E-03
Q3UHU8	*GTF2I*	General transcription factor II I	Thr623; Ser628	26.24	1.74E-02
Q9JKS4	*LDB3*	LIM domain binding 3	Thr119; Ser123	-16.36	5.98E-03
F6TFN2	*LMO7*	LIM domain only 7	Ser189	72.97	1.56E-06
Q14BP6	*LRRC74B*	Leucine rich repeat containing 74B	Ser253*; Ser260*	6.91	2.45E-02
P14873	*MAP1B*	Microtubule-associated protein 1B	Thr834; Ser$_{825-849}$	-11.36	4.32E-03
A0A0A0 MQC7	*MAPT*	Microtubule-associated protein tau	Ser688; Ser692; Ser696	-3.51	1.87E-02
Q9QVP4	*MYL7*	Myosin, light polypeptide 7, regulatory	Ser22	-59.72	3.68E-05
Q9JJW5	*MYOZ2*	Myozenin 2	Ser95; Ser116	-3.40	1.03E-02
Q9JJW5	*MYOZ2*	Myozenin 2	Thr111; Ser116; Ser/Thr/Tyr$_{92-132}$	6.30	3.41E-02
A0A087 WRY3	*NUCKS1*	Nuclear casein kinase and cyclin-dependent kinase substrate 1	Ser75; Ser79	-110.89	1.47E-03
A2AJ88	*PNPLA7*	Patatin-like phospholipase domain containing 7	Tyr283*; Thr286*	-6.39	2.33E-02
P63250	*KCNJ3*	Potassium inwardly-rectifying channel, subfamily J, member 3	Ser442	-3.16	1.39E-02
Q9ERE3	*SGK3*	Serum/glucocorticoid regulated kinase 3	Ser126; Ser129	-3.39	9.48E-04

Q61165	*SLC9A1*	Solute carrier family 9 (sodium/hydrogen exchanger), member 1	Ser609; Ser/Thr$_{601-616}$	-4.66	1.26E-02
A0A286YD34	*SORBS1*	Sorbin and SH3 domain containing 1	Ser15; Ser/Thr/Tyr$_{5-44}$	3.51	3.44E-02
E9PUK6	*SRRM1*	Serine/arginine repetitive matrix 1	Ser743; Thr745	-6.16	5.87E-04
E9PUK6	*SRRM1*	Serine/arginine repetitive matrix 1	Ser645; Ser647	-20.45	3.33E-03
E9PUK6	*SRRM1*	Serine/arginine repetitive matrix 1	Ser702; Ser704	-84.17	8.60E-03
Q8CH02	*SUGP1*	SURP and G patch domain containing 1	Thr128; Thr136	108.94	3.52E-03
Q8BWB1	*SYNPO2L*	Synaptopodin 2-like	Ser89; Ser/Thr$_{83-126}$	-3.45	3.99E-02
A2ASS6	*TTN*	Titin	Ser34063; Ser/Thr/Tyr$_{34062-34080}$	-11.48	4.75E-02
Q8VBT1	*TXLNB*	Taxilin beta	Ser467*; Ser/Thr$_{466-501}$	2.87	1.48E-02
A0A498WFS2	*UBXN1*	UBX domain protein 1	Ser/Thr$_{152-173}$	-12.03	1.57E-02
Q8K0L9	*ZBTB20*	Zinc finger and BTB domain containing 20	Thr695; Ser/Thr$_{680-700}$	-3.54	3.77E-02

Rows that contain multiple phosphorylation sites represent peptides that are doubly or triply phosphorylated. Ambiguous phosphorylation sites (with a probability <75%) are indicated by the range of amino acids corresponding to the identified peptide as a subscript. Amino acid numbering corresponds to the UniProt accession number listed with each protein. Phosphorylation sites marked with * indicate a novel phosphorylation site not previously annotated in PhosphoSitePlus (v. 6.6.0.4.). Ser, Serine; Thr, Threonine; Tyr, Tyrosine.
