# Supplementary material for: Alterations in cytoskeletal and Ca2+ cycling regulators in atria lacking the obscurin Ig58/59 module
Source: Front Cardiovasc Med. 2023 Apr 13;10:1085840. doi: 10.3389/fcvm.2023.1085840 (PMC10251194; doi:10.3389/fcvm.2023.1085840)
Supplement: Supplementary file 3 [file Table_3.pdf]

Supplemental Table 3. Proteins with significantly altered expression in *Obscn-ΔIg58/59* atria at 12-months.

Gene	Protein Name	Fold Change	P-value
ACADSB	Acyl-Coenzyme A dehydrogenase, short/branched chain	3.60	1.9E-08
C1QBP	Complement component 1, q subcomponent binding protein	1.78	5.7E-05
C4B	Complement component 4B (Chido blood group)	-1.77	1.0E-02
CD151	CD151 antigen	-1.76	1.9E-02
COX5A	Cytochrome c oxidase subunit 5A	-1.98	6.0E-05
EIF2S3X	Eukaryotic translation initiation factor 2, subunit 3, structural gene X-linked	-1.90	3.5E-03
EIF4G2	Eukaryotic translation initiation factor 4, gamma 2	1.68	1.2E-03
ELOC	Elongin C	-1.81	7.3E-04
FERMT3	Fermitin family member 3	1.89	1.2E-05
GLRX5	Glutaredoxin 5	2.03	1.5E-02
GOLT1B	Golgi transport 1B	1.91	9.4E-05
GRXCR1	Glutaredoxin, cysteine rich 1	1.80	1.7E-03
HDLBP	High density lipoprotein (HDL) binding protein	1.73	6.3E-03
HMOX2	Heme oxygenase 2	1.66	3.7E-03
IGHV1-26	Immunoglobulin heavy variable 1-26	4.27	1.5E-04
IGHV7-3	Immunoglobulin heavy variable 7-3	1.85	2.0E-02
IGKV3-2	Immunoglobulin kappa variable 3-2	-2.95	1.5E-05
IGKV8-27	Immunoglobulin kappa chain variable 8-27	-3.85	3.5E-05
KANK2	KN motif and ankyrin repeat domains 2	-1.82	7.8E-06
KRT1	Keratin 1	-2.35	1.3E-04
KRT5	Keratin 5	-16.44	3.1E-09
KRT6A	Keratin 6A	-5.39	6.4E-07
KRT14	Keratin 14	-29.48	3.4E-12
KRT16	Keratin 16	-10.18	1.9E-03
KRT17	Keratin 17	-9.51	3.1E-05
KRT42	Keratin 42	-7.17	3.0E-05
MCCC1	Methylcrotonoyl-Coenzyme A carboxylase 1 (alpha)	-2.05	1.3E-08
MLYCD	Malonyl-CoA decarboxylase	-1.87	1.9E-05
NAALAD2	N-acetylated alpha-linked acidic dipeptidase 2	-16.41	6.3E-11
OBSCN	Obscurin, cytoskeletal calmodulin and titin-interacting RhoGEF	-1.81	3.3E-03
PEBP1	Phosphatidylethanolamine binding protein 1	1.74	4.4E-06
PPID	Peptidylprolyl isomerase D (cyclophilin D)	-2.03	4.4E-05
PSMB3	Proteasome (prosome, macropain) subunit, beta type 3	-1.81	1.0E-02

PSMD4	Proteasome (prosome, macropain) 26S subunit, non-ATPase, 4	1.68	5.5E-03
RAB18	RAB18, member RAS oncogene family	1.91	1.5E-05
RAB8A	RAB8A, member RAS oncogene family	-1.93	1.7E-04
RHOC	Ras homolog family member C	-1.94	6.5E-03
RPLP2	Ribosomal protein, large P2	-1.68	2.6E-05
SAMHD1	SAM domain and HD domain, 1	-2.07	7.2E-05
SLC2A4	Solute carrier family 2 (facilitated glucose transporter), member 4	1.95	2.2E-07
SLMAP	Sarcolemma associated protein	-3.49	1.4E-03
SND1	Staphylococcal nuclease and tudor domain containing 1	2.05	1.4E-02
STARD10	START domain containing 10	-2.07	4.8E-02
STK24	Serine/threonine kinase 24	1.72	2.7E-03
TARS	Threonyl-tRNA synthetase	6.49	4.5E-08
TAX1BP3	Tax1 (human T cell leukemia virus type I) binding protein 3	1.80	1.9E-02
TNS1	Tensin 1	1.74	6.7E-06
WIPI1	WD repeat domain, phosphoinositide interacting 1	1.66	2.3E-05
