# Supplementary material for: Alterations in cytoskeletal and Ca2+ cycling regulators in atria lacking the obscurin Ig58/59 module
Source: Front Cardiovasc Med. 2023 Apr 13;10:1085840. doi: 10.3389/fcvm.2023.1085840 (PMC10251194; doi:10.3389/fcvm.2023.1085840)
Supplement: Supplementary file 4 [file Table_4.pdf]

Supplemental Table 4. Proteins with significantly altered phosphorylation in *Obscn-ΔIg58/59* atria at 12-months.

UniProt Accession	Gene	Protein Name	Site	Fold Change	P-value
Q3UHJ0	*AAK1*	AP2 associated kinase 1	Ser/Tyr_{10-37}	2.27	1.03E-02
Q9QYC0	*ADD1*	Adducin 1 (alpha)	Thr614; $Ser/Thr_{598-629}$	2.82	2.26E-03
O54774	*AP3D1*	Adaptor-related protein complex 3, delta 1 subunit	$Ser/Thr_{859-895}$	4.01	2.50E-04
O35841	*API5*	Apoptosis inhibitor 5	Ser464	-2.43	4.37E-02
Q9WV35	*APOBEC2*	Apolipoprotein B mRNA editing enzyme, catalytic polypeptide 2	Ser15	3.86	2.36E-02
O55143	*ATP2A2*	ATPase, Ca++ transporting, cardiac muscle, slow twitch 2	$Ser/Thr/Tyr_{372-397}$	-3.43	7.80E-03
Q6PHZ2	*CAMK2D*	Calcium/calmodulin-dependent protein kinase II, delta	$Ser/Thr_{323-344}$	2.31	3.25E-02
Q6PHZ2	*CAMK2D*	Calcium/calmodulin-dependent protein kinase II, delta	Thr331	2.57	9.26E-03
P51125	*CAST*	Calpastatin	Ser219	-4.32	1.20E-02
O54724	*CAVIN1*	Caveolae associated 1	Ser204; Ser205	3.22	2.22E-02
Q63918	*CAVIN2*	Caveolae associated 2	Ser204; $Ser/Thr_{192-227}$	-6.29	4.03E-03
Q63918	*CAVIN2*	Caveolae associated 2	Ser359	3.14	1.74E-03
Q63918	*CAVIN2*	Caveolae associated 2	Ser218; $Ser/Thr_{192-222}$	3.78	3.75E-07
Q63918	*CAVIN2*	Caveolae associated 2	Ser363	13.57	1.11E-05
E9QLJ0	*CMYA5*	Cardiomyopathy associated 5 (myospryn)	Ser155	-2.17	3.27E-04
E9QLJ0	*CMYA5*	Cardiomyopathy associated 5 (myospryn)	Ser705	2.31	4.36E-02
Q60598	*CTTN*	Cortactin	Thr401; Ser405; Ser407	-5.94	4.38E-06
P11531	*DMD*	Dystrophin, muscular dystrophy	$Ser/Thr_{3624-3664}$	-3.56	3.08E-03
Q9WV69	*DMTN*	Dematin actin binding protein	Ser226	2.98	2.60E-02
E9QP49	*EHBP1L1*	EH domain binding protein 1-like 1	Thr164*	2.45	1.27E-03
Q61554	*FBN1*	Fibrillin 1	Ser2704	-2.81	1.05E-02
Q61554	*FBN1*	Fibrillin 1	Ser2705	3.40	6.44E-05
Q9DB70	*FUNDC1*	FUN14 domain containing 1	Ser13	-9.45	1.20E-03

Q6P5B5	*FXR2*	Fragile X mental retardation, autosomal homolog 2	Ser535	-3.56	2.83E-05
P23242	*GJA1*	Gap junction protein, alpha 1 (connexin-43)	Ser/Thr$_{320-345}$	-2.97	2.21E-02
P23242	*GJA1*	Gap junction protein, alpha 1 (connexin-43)	Ser325; Thr326	8.22	4.17E-02
Q9JLM9	*GRB14*	Growth factor receptor bound protein 14	Ser373	2.34	2.41E-03
Q9Z1E4	*GYS1*	Glycogen synthase 1, muscle	Ser728	-2.49	1.47E-02
A0A0R4J008	*HDAC2*	Histone deacetylase 2	Ser394	-6.24	3.15E-03
P18608	*HMGN1*	High mobility group nucleosomal binding domain 1	Ser87	2.38	3.93E-02
Q8VDM6	*HNRNPUL1*	Heterogeneous nuclear ribonucleoprotein U-like 1	Ser721	-2.27	2.17E-05
Q3TEA8	*HP1BP3*	Heterochromatin protein 1, binding protein 3	Thr85	-2.28	4.75E-02
G5E8J6	*HRC*	Histidine rich calcium binding protein	Ser272	-2.66	5.28E-03
Q3U2G2	*HSPA4*	Heat shock protein 4 (70 kDa)	Thr540	-3.87	6.20E-05
E9Q0J5	*KIF21A*	Kinesin family member 21A	Ser/Thr$_{853-883}$	2.43	8.82E-03
Q9QXD8	*LIMD1*	LIM domains containing 1	Ser417*	-2.30	1.08E-02
P63085	*MAPK1*	Mitogen-activated protein kinase 1	Thr183; Thr188	2.35	2.03E-02
P47811	*MAPK14*	Mitogen-activated protein kinase 14	Thr185	-2.43	2.55E-03
Q3UIK0	*MYBPC3*	Myosin binding protein C, cardiac	Ser268*	-2.53	3.92E-03
Q9QVP4	*MYL7*	Myosin, light polypeptide 7, regulatory	Ser23	-2.71	2.89E-02
Q3UIZ8	*MYLK3*	Myosin light chain kinase 3	Ser155	-5.92	2.16E-02
Q9JJW5	*MYOZ2*	Myozenin 2	Thr107	-4.09	1.47E-02
Q80XU3	*NUCKS1*	Nuclear casein kinase and cyclin-dependent kinase substrate 1	Ser79	2.85	2.04E-02
Q80XU3	*NUCKS1*	Nuclear casein kinase and cyclin-dependent kinase substrate 1	Ser79	2.91	1.75E-02
Q5QNQ6	*OSPB2*	Oxysterol binding protein 2	Ser281	2.87	5.50E-04

Q9ET54	*PALLD*	Palladin, cytoskeletal associated protein	Ser1009	12.90	5.09E-08
P49586	*PCYT1A*	Phosphate cytidylyltransferase 1, choline, alpha isoform	Ser/Thr$_{318-328}$	2.72	1.53E-03
Q80UU9	*PGRMC2*	Progesterone receptor membrane component 2	Thr205	2.48	1.08E-02
Q9QXS1	*PLEC*	Plectin	Ser4415	2.84	5.83E-04
Q8C8U0	*PPFIBP1*	PTPRF interacting protein, binding protein 1 (liprin beta 1)	Ser541	3.73	9.15E-04
Q8BXN7	*PPM1K*	Protein phosphatase 1K, (PP2C domain containing)	Ser248	-2.83	1.39E-04
Q7TSI3	*PPP6R1*	Protein phosphatase 6, regulatory subunit 1	Ser662	-2.84	7.67E-03
Q5U3K5	*RABL6*	RAB, member RAS oncogene family-like 6	Ser594; Thr597	-3.75	7.15E-07
A0A1W2 P712	*RALGAPA1*	Ral GTPase activating protein, alpha subunit 1	Ser772	2.58	4.85E-06
Q61193	*RGL2*	Ral guanine nucleotide dissociation stimulator-like 2	Ser617	-3.33	7.44E-05
E9Q401	*RYR2*	Ryanodine receptor 2, cardiac	Ser2810	3.52	7.81E-04
F8WJE0	*SAMHD1*	SAM domain and HD domain, 1	Thr52	4.47	1.53E-05
Q64213	*SF1*	Splicing factor 1	Ser80	2.26	1.65E-02
Q62417	*SORBS1*	Sorbin and SH3 domain containing 1	Thr409	2.97	3.70E-03
E9QQ25	*SPEG*	SPEG complex locus	Ser2200*	-7.06	3.89E-03
E9QQ25	*SPEG*	SPEG complex locus	Ser2182	-3.74	4.62E-03
Q52KI8	*SRRM1*	Serine/arginine repetitive matrix 1	Thr913	-2.73	1.18E-02
Q8BTI8	*SRRM2*	Serine/arginine repetitive matrix 2	Ser1216	-2.63	3.28E-05
Q70IV5	*SYNM*	Synemin, intermediate filament protein	Ser1087	2.77	7.53E-05
E9Q1U2	*SYNPO2*	Synaptopodin 2	Ser629	58.93	1.72E-09
B2RQK7	*SYNPO2L*	Synaptopodin 2-like	Thr88*; Ser97	4.39	7.66E-03
Q5SVR0	*TBC1D9B*	TBC1 domain family, member 9B	Ser/Thr$_{411-458}$	-4.62	6.32E-03
O70548	*TCAP*	Titin-cap	Ser161	-2.23	2.32E-02
Q9ERA6	*TFIP11*	Tuftelin interacting protein 11	Ser211	-2.73	6.43E-03
P24529	*TH*	Tyrosine hydroxylase	Thr30	-6.58	1.20E-06
Q61029	*TMPO*	Thymopoietin	Ser179	2.34	4.24E-02

E9Q0S6	*TNS1*	Tensin 1	Ser1451	-2.71	4.47E-02
Q64511	*TOP2B*	Topoisomerase (DNA) II beta	Ser1387	5.19	1.68E-03
A2ASS6	*TTN*	Titin	Ser33875; Ser33880	2.34	9.82E-03
A2ASS6	*TTN*	Titin	Ser9459	4.52	1.25E-03
A2ASS6	*TTN*	Titin	Ser34470	6.20	1.05E-04
Q64727	*VCL*	Vinculin	Ser721	10.97	1.25E-04
Q4U4S6	*XIRP2*	Xin actin-binding repeat containing 2	Ser2031*	-2.88	6.90E-04

Rows that contain multiple phosphorylation sites represent peptides that are doubly or triply phosphorylated. Ambiguous phosphorylation sites (with a probability <75%) are indicated by the range of amino acids corresponding to the identified peptide as a subscript. Amino acid numbering corresponds to the UniProt accession number listed with each protein. Phosphorylation sites marked with * indicate a novel phosphorylation site not previously annotated in PhosphoSitePlus (v. 6.6.0.4.). Ser, Serine; Thr, Threonine; Tyr, Tyrosine.
