# Supplementary material for: Alterations in cytoskeletal and Ca2+ cycling regulators in atria lacking the obscurin Ig58/59 module
Source: Front Cardiovasc Med. 2023 Apr 13;10:1085840. doi: 10.3389/fcvm.2023.1085840 (PMC10251194; doi:10.3389/fcvm.2023.1085840)
Supplement: Supplementary file 5 [file Table_5.pdf]

Supplemental Table 5. Molecular pathways and cellular functions associated with significantly altered proteins in *Obscn-ΔIg58/59* atria at 6-months.

Molecular Pathways	
D-myo-GJA (1,4,5,6)-Tetrakisphosphate Biosynthesis (1.64)	
ACP6	Acid phosphatase 6, lysophosphatidic
CAR3	Carbonic anhydrase 3
NUDT4	Nudix (nucleoside diphosphate linked moiety X)-type motif 4
D-myo-inositol (3,4,5,6)-tetrakisphosphate Biosynthesis (1.64)	
ACP6	Acid phosphatase 6, lysophosphatidic
CAR3	Carbonic anhydrase 3
NUDT4	Nudix (nucleoside diphosphate linked moiety X)-type motif 4
3-phosphoinositide Degradation (1.60)	
ACP6	Acid phosphatase 6, lysophosphatidic
CAR3	Carbonic anhydrase 3
NUDT4	Nudix (nucleoside diphosphate linked moiety X)-type motif 4
D-myo-inositol-5-phosphate Metabolism (1.55)	
ACP6	Acid phosphatase 6, lysophosphatidic
CAR3	Carbonic anhydrase 3
NUDT4	Nudix (nucleoside diphosphate linked moiety X)-type motif 4
3-phosphoinositide Biosynthesis (1.51)	
ACP6	Acid phosphatase 6, lysophosphatidic
CAR3	Carbonic anhydrase 3
NUDT4	Nudix (nucleoside diphosphate linked moiety X)-type motif 4
Superpathway of Inositol Phosphate Compounds (1.40)	
ACP6	Acid phosphatase 6, lysophosphatidic
CAR3	Carbonic anhydrase 3
NUDT4	Nudix (nucleoside diphosphate linked moiety X)-type motif 4
Cellular Functions	
Permeabilization of mitochondria (2.73)	
BCL2L13	BCL2-like 13 (apoptosis facilitator)
HK2	Hexokinase 2
PDIA3	Protein disulfide isomerase associated 3
Shape change of fibroblast cell lines (1.69)	
CD151	CD151 antigen
CORO1B	Coronin, actin binding protein 1B
SORBS1	Sorbin and SH3 domain containing 1
Termination of translation of protein (1.64)	
MRPL10	Mitochondrial ribosomal protein L10
MRPS28	Mitochondrial ribosomal protein S28
UPF1	UPF1 regulator of nonsense transcripts homolog (yeast)
Phagocytosis of myeloid cells (1.60)	
GLRX	Glutaredoxin
MFGE8	Milk fat globule EGF and factor V/VIII domain containing (lactadherin)
NHLRC2	NHL repeat containing 2
Transport of monosaccharide (1.51)	

HK2	Hexokinase 2
SORBS1	Sorbin and SH3 domain containing 1
STX16	Syntaxin 16
Phagocytosis of phagocytes (1.51)	
GLRX	Glutaredoxin
MFGE8	Milk fat globule EGF and factor V/VIII domain containing (lactadherin)
NHLRC2	NHL repeat containing 2
Cell viability of neurons (1.40)	
HK2	Hexokinase 2
PDIA3	Protein disulfide isomerase associated 3
UPF1	UPF1 regulator of nonsense transcripts homolog (yeast)
Permeability of vasculature (1.37)	
CAMK2D	Calcium/calmodulin-dependent protein kinase II, delta
CD151	CD151 antigen
MFGE8	Milk fat globule EGF and factor V/VIII domain containing (lactadherin)

Significantly altered proteins and their corresponding gene symbols are listed under the molecular pathway and cellular functions they are associated with. The p-value for each molecular pathway and cellular function is represented as $-Log_{10}$(p-value).
