# Supplementary material for: Alterations in cytoskeletal and Ca2+ cycling regulators in atria lacking the obscurin Ig58/59 module
Source: Front Cardiovasc Med. 2023 Apr 13;10:1085840. doi: 10.3389/fcvm.2023.1085840 (PMC10251194; doi:10.3389/fcvm.2023.1085840)
Supplement: Supplementary file 6 [file Table_6.pdf]

Supplemental Table 6. Molecular pathways and cellular functions associated with significantly altered phospho-proteins in *Obscn-ΔIg58/59* atria at 6-months.

Molecular Pathways	
Apelin Cardiomyocyte Signaling Pathway (1.82)	
ATP2A3	ATPase, Ca++ transporting, ubiquitous
MYL7	Myosin, light polypeptide 7, regulatory
SLC9A1	Solute carrier family 9 (sodium/hydrogen exchanger), member 1
Cellular Functions	
Organization of sarcomere (3.64)	
FHOD3	Formin homology 2 domain containing 3
LDB3	LIM domain binding 3
MYOZ2	Myozenin 2
SYNPO2L	Synaptopodin 2-like
TTN	Titin
Disassembly of filaments (3.39)	
ABCC1	ATP-binding cassette, sub-family C (CFTR/MRP), member 1
MAP1B	Microtubule-associated protein 1B
MAPT	Microtubule-associated protein tau
SLC9A1	Solute carrier family 9 (sodium/hydrogen exchanger), member 1
Organization of cells (2.97)	
FHOD3	Formin homology 2 domain containing 3
LDB3	LIM domain binding 3
MAPT	Microtubule-associated protein tau
MYOZ2	Myozenin 2
SYNPO2L	Synaptopodin 2-like
TTN	Titin
Depolymerization of microtubules (2.77)	
ABCC1	ATP-binding cassette, sub-family C (CFTR/MRP), member 1
MAP1B	Microtubule-associated protein 1B
MAPT	Microtubule-associated protein tau
Bundling of filaments (2.77)	
MAP1B	Microtubule-associated protein 1B
MAPT	Microtubule-associated protein tau
TTN	Titin
Elongation of neurites (2.77)	
MAP1B	Microtubule-associated protein 1B
MAPT	Microtubule-associated protein tau
SLC9A1	Solute carrier family 9 (sodium/hydrogen exchanger), member 1
Transport of ion (2.72)	
ABCC1	ATP-binding cassette, sub-family C (CFTR/MRP), member 1
ATP2A3	ATPase, Ca++ transporting, ubiquitous
CLNS1A	Chloride channel, nucleotide-sensitive, 1A
KCNJ3	Potassium inwardly-rectifying channel, subfamily J, member 3
SGK3	Serum/glucocorticoid regulated kinase 3
SLC9A1	Solute carrier family 9 (sodium/hydrogen exchanger), member 1

Hypertrophy of cardiac muscle (2.04)	
FHOD3	Formin homology 2 domain containing 3
MYOZ2	Myozenin 2
SLC9A1	Solute carrier family 9 (sodium/hydrogen exchanger), member 1
TTN	Titin
Quantity of filaments (2.03)	
MAP1B	Microtubule-associated protein 1B
MAPT	Microtubule-associated protein tau
SLC9A1	Solute carrier family 9 (sodium/hydrogen exchanger), member 1
TTN	Titin
Fibrogenesis (2.02)	
FHOD3	Formin homology 2 domain containing 3
LDB3	LIM domain binding 3
MAP1B	Microtubule-associated protein 1B
MAPT	Microtubule-associated protein tau
MYOZ2	Myozenin 2
SLC9A1	Solute carrier family 9 (sodium/hydrogen exchanger), member 1
SYNPO2L	Synaptopodin 2-like
TTN	Titin

Significantly altered phospho-proteins and their corresponding gene symbols are listed under the molecular pathway and cellular functions they are associated with. The p-value for each molecular pathway and cellular function is represented as $-Log_{10}$(p-value).
