# Supplementary material for: Alterations in cytoskeletal and Ca2+ cycling regulators in atria lacking the obscurin Ig58/59 module
Source: Front Cardiovasc Med. 2023 Apr 13;10:1085840. doi: 10.3389/fcvm.2023.1085840 (PMC10251194; doi:10.3389/fcvm.2023.1085840)
Supplement: Supplementary file 7 [file Table_7.pdf]

Supplemental Table 7. Molecular pathways and cellular functions associated with significantly altered proteins in *Obscn-ΔIg58/59* atria at 12-months.

Molecular Pathways	
Communication between Innate and Adaptive Immune Cells (2.20)	
IGHV1-26	Immunoglobulin heavy variable 1-26
IGHV7-3	Immunoglobulin heavy variable 7-3
IGKV3-2	Immunoglobulin kappa variable 3-2
IGKV8-27	Immunoglobulin kappa chain variable 8-27
IL-15 Signaling (1.71)	
IGHV1-26	Immunoglobulin heavy variable 1-26
IGHV7-3	Immunoglobulin heavy variable 7-3
IGKV3-2	Immunoglobulin kappa variable 3-2
IGKV8-27	Immunoglobulin kappa chain variable 8-27
Systemic Lupus Erythematosus In B Cell Signaling Pathway (1.47)	
IGHV1-26	Immunoglobulin heavy variable 1-26
IGHV7-3	Immunoglobulin heavy variable 7-3
IGKV3-2	Immunoglobulin kappa variable 3-2
IGKV8-27	Immunoglobulin kappa chain variable 8-27
B Cell Receptor Signaling (1.45)	
IGHV1-26	Immunoglobulin heavy variable 1-26
IGHV7-3	Immunoglobulin heavy variable 7-3
IGKV3-2	Immunoglobulin kappa variable 3-2
IGKV8-27	Immunoglobulin kappa chain variable 8-27
Cellular Functions	
Keratinization (5.69)	
KRT1	Keratin 1
KRT5	Keratin 5
KRT6A	Keratin 6A
KRT14	Keratin 14
KRT16	Keratin 16
KRT17	Keratin 17
Chronic fatigue syndrome (3.91)	
C4B	Complement component 4B (Chido blood group)
KRT14	Keratin 14
KRT16	Keratin 16
KRT17	Keratin 17
Morphology of keratinocytes (3.61)	
KRT14	Keratin 14
KRT16	Keratin 16
KRT6A	Keratin 6A
Permeability of blood vessel (1.90)	
C4B	Complement component 4B (Chido blood group)
CD151	CD151 antigen
KRT1	Keratin 1

Morphogenesis of epithelial tissue (1.79)	
KRT16	Keratin 16
KRT6A	Keratin 6A
KRT17	Keratin 17
Invasion of cells (1.55)	
C1QBP	Complement component 1, q subcomponent binding protein
CD151	CD151 antigen
FERMT3	Fermitin family member 3
HDLBP	High density lipoprotein (HDL) binding protein
KRT14	Keratin 14
KRT17	Keratin 17
OBSCN	Obscurin
PEBP1	Phosphatidylethanolamine binding protein 1
RHOC	Ras homolog family member C
STK24	Serine/threonine kinase 24
TNS1	Tensin 1
Binding of endothelial cells (1.50)	
C1QBP	Complement component 1, q subcomponent binding protein
CD151	CD151 antigen
FERMT3	Fermitin family member 3
KRT1	Keratin 1
Beta oxidation of fatty acid (1.48)	
ACADSB	Acyl-Coenzyme A dehydrogenase, short/branched chain
MLYCD	Malonyl-CoA decarboxylase
PEBP1	Phosphatidylethanolamine binding protein 1
Small GTPase mediated signal transduction (1.47)	
KANK2	KN motif and ankyrin repeat domains 2
OBSCN	Obscurin
RHOC	Ras homolog family member C
TAX1BP3	Tax1 (human T cell leukemia virus type I) binding protein 3
Inflammation of organ (1.36)	
C4B	Complement component 4B (Chido blood group)
CD151	CD151 antigen
GLRX	Glutaredoxin
HMOX2	Heme oxygenase 2
KRT1	Keratin 1
KRT14	Keratin 14
KRT16	Keratin 16
KRT17	Keratin 17
KRT5	Keratin 5
PPID	Peptidylprolyl isomerase D (cyclophilin D)
SLC2A4	Solute carrier family 2 (facilitated glucose transporter), member 4
TNS1	Tensin 1

Significantly altered proteins and their corresponding gene symbols are listed under the molecular pathway and cellular functions they are associated with. The p-value for each molecular pathway and cellular function is represented as -Log_{10}(p-value).
