# Supplementary material for: Alterations in cytoskeletal and Ca2+ cycling regulators in atria lacking the obscurin Ig58/59 module
Source: Front Cardiovasc Med. 2023 Apr 13;10:1085840. doi: 10.3389/fcvm.2023.1085840 (PMC10251194; doi:10.3389/fcvm.2023.1085840)
Supplement: Supplementary file 8 [file Table_8.pdf]

Supplemental Table 8. Molecular pathways and cellular functions associated with significantly altered phospho-proteins in *Obscn-ΔIg58/59* atria at 12-months.

Molecular Pathways	
Dilated Cardiomyopathy Signaling Pathway (2.63)	
ATP2A2	ATPase, Ca++ transporting, cardiac muscle, slow twitch 2
CAMK2D	Calcium/calmodulin-dependent protein kinase II, delta
DMD	Dystrophin, muscular dystrophy
MAPK1	Mitogen-activated protein kinase 1
MAPK14	Mitogen-activated protein kinase 14
MYBPC3	Myosin binding protein C, cardiac
MYL7	Myosin, light polypeptide 7, regulatory
RYR2	Ryanodine receptor 2, cardiac
TTN	Titin
Integrin Signaling (1.86)	
CTTN	Cortactin
MAPK1	Mitogen-activated protein kinase 1
MYL7	Myosin, light polypeptide 7, regulatory
MYLK3	Myosin light chain kinase 3
TTN	Titin
VCL	Vinculin
Protein Kinase A Signaling (1.71)	
ADD1	Adducin 1 (alpha)
CAMK2D	Calcium/calmodulin-dependent protein kinase II, delta
GYS1	Glycogen synthase 1, muscle
MAPK1	Mitogen-activated protein kinase 1
MYL7	Myosin, light polypeptide 7, regulatory
MYLK3	Myosin light chain kinase 3
RYR2	Ryanodine receptor 2, cardiac
TH	Tyrosine hydroxylase
TTN	Titin
BAG2 Signaling Pathway (1.69)	
HSPA4	Heat shock protein 4 (70 kDa)
MAPK1	Mitogen-activated protein kinase 1
MAPK14	Mitogen-activated protein kinase 14
Role of PKR in Interferon Induction and Antiviral Response (1.57)	
HSPA4	Heat shock protein 4 (70 kDa)
MAPK1	Mitogen-activated protein kinase 1
MAPK14	Mitogen-activated protein kinase 14
Apelin Cardiomyocyte Signaling Pathway (1.50)	
ATP2A2	ATPase, Ca++ transporting, cardiac muscle, slow twitch 2
MAPK1	Mitogen-activated protein kinase 1
MAPK14	Mitogen-activated protein kinase 14
MYL7	Myosin, light polypeptide 7, regulatory
Regulation of Cellular Mechanics by Calpain Protease (1.46)	
CAST	Calpastatin

MAPK1	Mitogen-activated protein kinase 1
VCL	Vinculin
Coronavirus Pathogenesis Pathway (1.46)	
HDAC2	Histone deacetylase 2
MAPK1	Mitogen-activated protein kinase 1
MAPK14	Mitogen-activated protein kinase 14
Calcium Signaling (1.42)	
ATP2A2	ATPase, Ca++ transporting, cardiac muscle, slow twitch 2
CAMK2D	Calcium/calmodulin-dependent protein kinase II, delta
HDAC2	Histone deacetylase 2
MAPK1	Mitogen-activated protein kinase 1
MYL7	Myosin, light polypeptide 7, regulatory
RYR2	Ryanodine receptor 2, cardiac
CDC42 Signaling (1.37)	
MAPK1	Mitogen-activated protein kinase 1
MAPK14	Mitogen-activated protein kinase 14
MYL7	Myosin, light polypeptide 7, regulatory
Cellular Functions	
Cardiogenesis (5.27)	
ATP2A2	ATPase, Ca++ transporting, cardiac muscle, slow twitch 2
CAMK2D	Calcium/calmodulin-dependent protein kinase II, delta
DMD	Dystrophin, muscular dystrophy
FBN1	Fibrillin 1
GJA1	Gap junction protein, alpha 1 (connexin-43)
GYS1	Glycogen synthase 1, muscle
HDAC2	Histone deacetylase 2
MAPK1	Mitogen-activated protein kinase 1
MAPK14	Mitogen-activated protein kinase 14
MYBPC3	Myosin binding protein C, cardiac
MYL7	Myosin, light polypeptide 7, regulatory
MYLK3	Myosin light chain kinase 3
MYOZ2	Myozenin 2
PLEC	Plectin
SPEG	SPEG complex locus
SYNPO2L	Synaptopodin 2-like
TCAP	Titin-cap
TH	Tyrosine hydroxylase
TTN	Titin
VCL	Vinculin
XIRP2	Xin actin-binding repeat containing 2
Morphology of cardiomyocytes (4.92)	
ATP2A2	ATPase, Ca++ transporting, cardiac muscle, slow twitch 2
CAMK2D	Calcium/calmodulin-dependent protein kinase II, delta
DMD	Dystrophin, muscular dystrophy
HDAC2	Histone deacetylase 2

MAPK1	Mitogen-activated protein kinase 1
MAPK14	Mitogen-activated protein kinase 14
MYBPC3	Myosin binding protein C, cardiac
MYLK3	Myosin light chain kinase 3
MYOZ2	Myozenin 2
PLEC	Plectin
RYR2	Ryanodine receptor 2, cardiac
TH	Tyrosine hydroxylase
TTN	Titin
VCL	Vinculin
Development of striated muscle (4.90)	
DMD	Dystrophin, muscular dystrophy
GJA1	Gap junction protein, alpha 1 (connexin-43)
HDAC2	Histone deacetylase 2
MAPK14	Mitogen-activated protein kinase 14
MYBPC3	Myosin binding protein C, cardiac
MYLK3	Myosin light chain kinase 3
MYOZ2	Myozenin 2
PLEC	Plectin
SPEG	SPEG complex locus
TCAP	Titin-cap
TTN	Titin
VCL	Vinculin
XIRP2	Xin actin-binding repeat containing 2
Muscular hypertrophy (4.61)	
CAMK2D	Calcium/calmodulin-dependent protein kinase II, delta
CAST	Calpastatin
DMD	Dystrophin, muscular dystrophy
HDAC2	Histone deacetylase 2
MAPK1	Mitogen-activated protein kinase 1
MAPK14	Mitogen-activated protein kinase 14
MYBPC3	Myosin binding protein C, cardiac
MYOZ2	Myozenin 2
RYR2	Ryanodine receptor 2, cardiac
SPEG	SPEG complex locus
TCAP	Titin-cap
TTN	Titin
Morphology of muscle (4.54)	
APOBEC2	Apolipoprotein B mRNA editing enzyme, catalytic polypeptide 2
ATP2A2	ATPase, Ca++ transporting, cardiac muscle, slow twitch 2
CAMK2D	Calcium/calmodulin-dependent protein kinase II, delta
CAST	Calpastatin
DMD	Dystrophin, muscular dystrophy
FBN1	Fibrillin 1
HDAC2	Histone deacetylase 2
HRC	Histidine rich calcium binding protein

MAPK1	Mitogen-activated protein kinase 1
MAPK14	Mitogen-activated protein kinase 14
MYBPC3	Myosin binding protein C, cardiac
MYLK3	Myosin light chain kinase 3
MYOZ2	Myozenin 2
PLEC	Plectin
RYR2	Ryanodine receptor 2, cardiac
SPEG	SPEG complex locus
SYNM	Synemin, intermediate filament protein
TCAP	Titin-cap
TH	Tyrosine hydroxylase
TTN	Titin
VCL	Vinculin
Formation of muscle cells (4.34)	
CAMK2D	Calcium/calmodulin-dependent protein kinase II, delta
DMD	Dystrophin, muscular dystrophy
HDAC2	Histone deacetylase 2
MAPK14	Mitogen-activated protein kinase 14
MYBPC3	Myosin binding protein C, cardiac
MYLK3	Myosin light chain kinase 3
MYOZ2	Myozenin 2
PLEC	Plectin
SPEG	SPEG complex locus
SYNPO2L	Synaptopodin 2-like
TCAP	Titin-cap
TTN	Titin
Contraction of heart (3.48)	
ATP2A2	ATPase, Ca++ transporting, cardiac muscle, slow twitch 2
CAMK2D	Calcium/calmodulin-dependent protein kinase II, delta
DMD	Dystrophin, muscular dystrophy
GJA1	Gap junction protein, alpha 1 (connexin-43)
HRC	Histidine rich calcium binding protein
MYBPC3	Myosin binding protein C, cardiac
MYL7	Myosin, light polypeptide 7, regulatory
RYR2	Ryanodine receptor 2, cardiac
TCAP	Titin-cap
TH	Tyrosine hydroxylase
TTN	Titin
Familial arrhythmogenic right ventricular dysplasia type 1 (3.37)	
MYBPC3	Myosin binding protein C, cardiac
PLEC	Plectin
RYR2	Ryanodine receptor 2, cardiac
TTN	Titin
Fibrogenesis (3.32)	
ADD1	Adducin 1 (alpha)
CAMK2D	Calcium/calmodulin-dependent protein kinase II, delta

CTTN	Cortactin
DMD	Dystrophin, muscular dystrophy
FBN1	Fibrillin 1
MAPK1	Mitogen-activated protein kinase 1
MAPK14	Mitogen-activated protein kinase 14
MYBPC3	Myosin binding protein C, cardiac
MYLK3	Myosin light chain kinase 3
MYOZ2	Myozenin 2
PALLD	Palladin, cytoskeletal associated protein
SORBS1	Sorbin and SH3 domain containing 1
SYNM	Synemin, intermediate filament protein
SYNPO2L	Synaptopodin 2-like
TCAP	Titin-cap
TNS1	Tensin 1
TTN	Titin
Intrinsic cardiomyopathy (3.20)	
ATP2A2	ATPase, Ca++ transporting, cardiac muscle, slow twitch 2
CAMK2D	Calcium/calmodulin-dependent protein kinase II, delta
CAST	Calpastatin
DMD	Dystrophin, muscular dystrophy
GJA1	Gap junction protein, alpha 1 (connexin-43)
MYBPC3	Myosin binding protein C, cardiac
MYOZ2	Myozenin 2
PLEC	Plectin
RYR2	Ryanodine receptor 2, cardiac
SPEG	SPEG complex locus
TCAP	Titin-cap
TMPO	Thymopoietin
TOP2B	Topoisomerase (DNA) II beta
TTN	Titin
VCL	Vinculin

Significantly altered phospho-proteins and their corresponding gene symbols are listed under the molecular pathway and cellular functions they are associated with. The p-value for each molecular pathway and cellular function is represented as $-Log_{10}$(p-value).
