# Supplementary material for: Alterations in cytoskeletal and Ca2+ cycling regulators in atria lacking the obscurin Ig58/59 module
Source: Front Cardiovasc Med. 2023 Apr 13;10:1085840. doi: 10.3389/fcvm.2023.1085840 (PMC10251194; doi:10.3389/fcvm.2023.1085840)
Supplement: Supplementary file 17 [file Image_1.pdf]

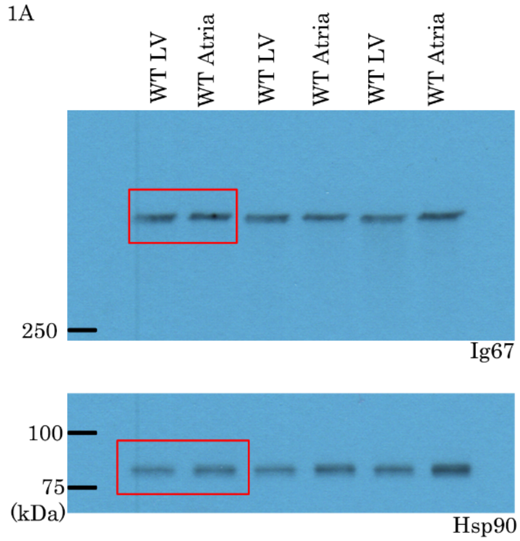

Original blots in Fig. 1A

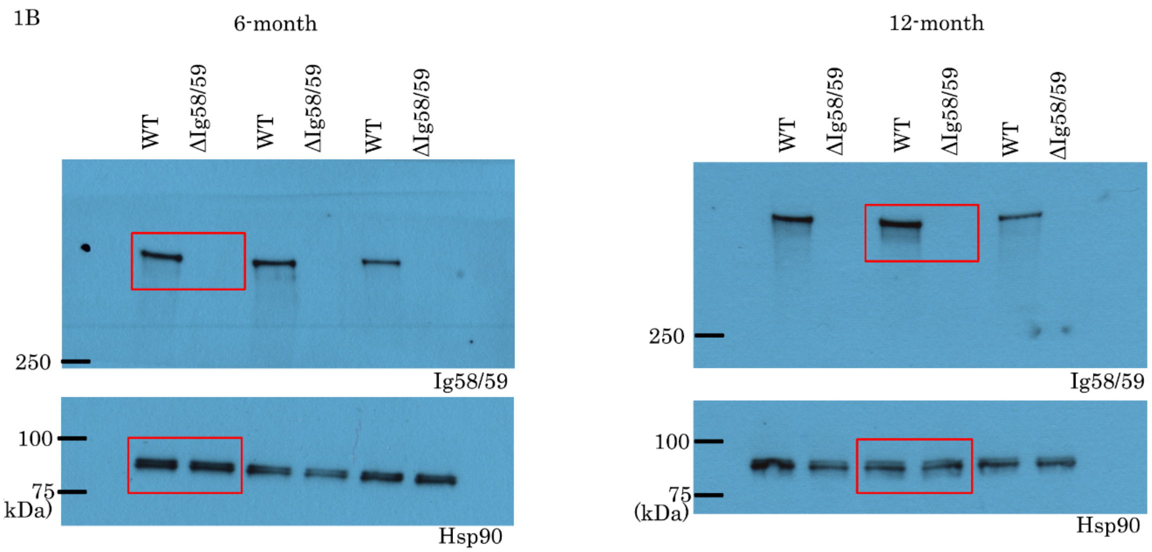

Original blots in Fig. 1B

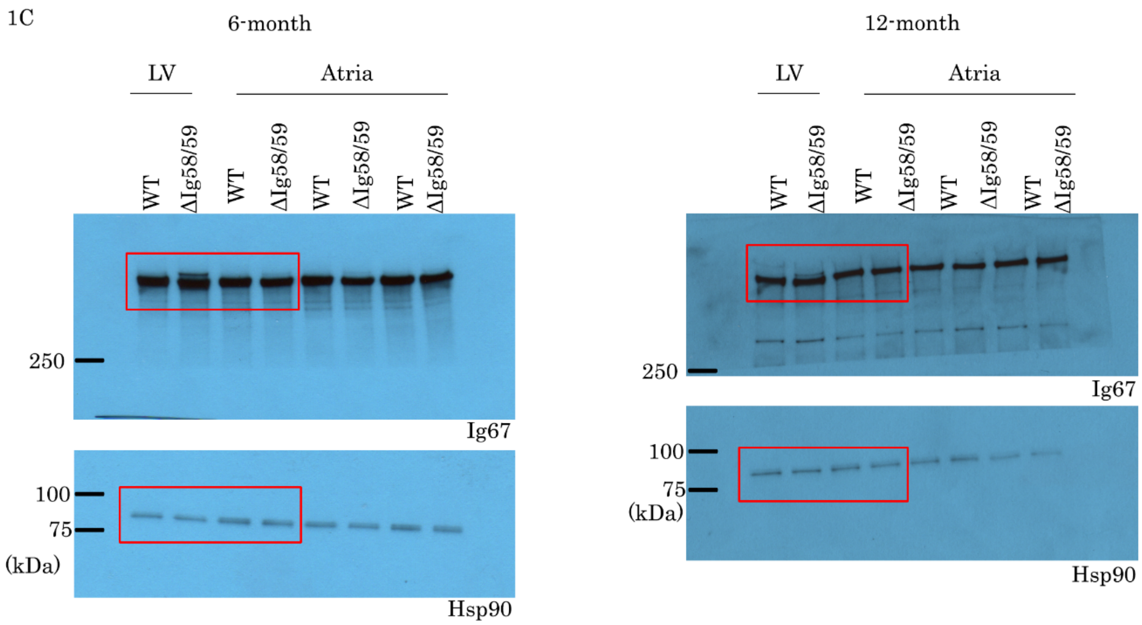

Original blots in Fig. 1C

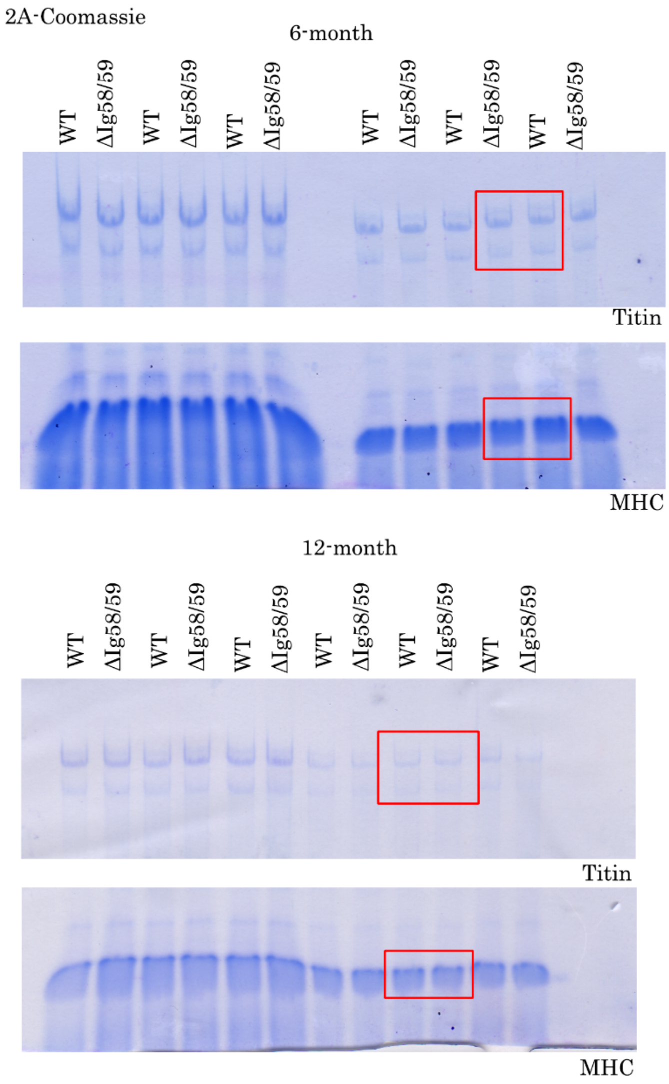

Original Coomassie Gels in Fig. 2A

2A- 6mo-Immunoblot

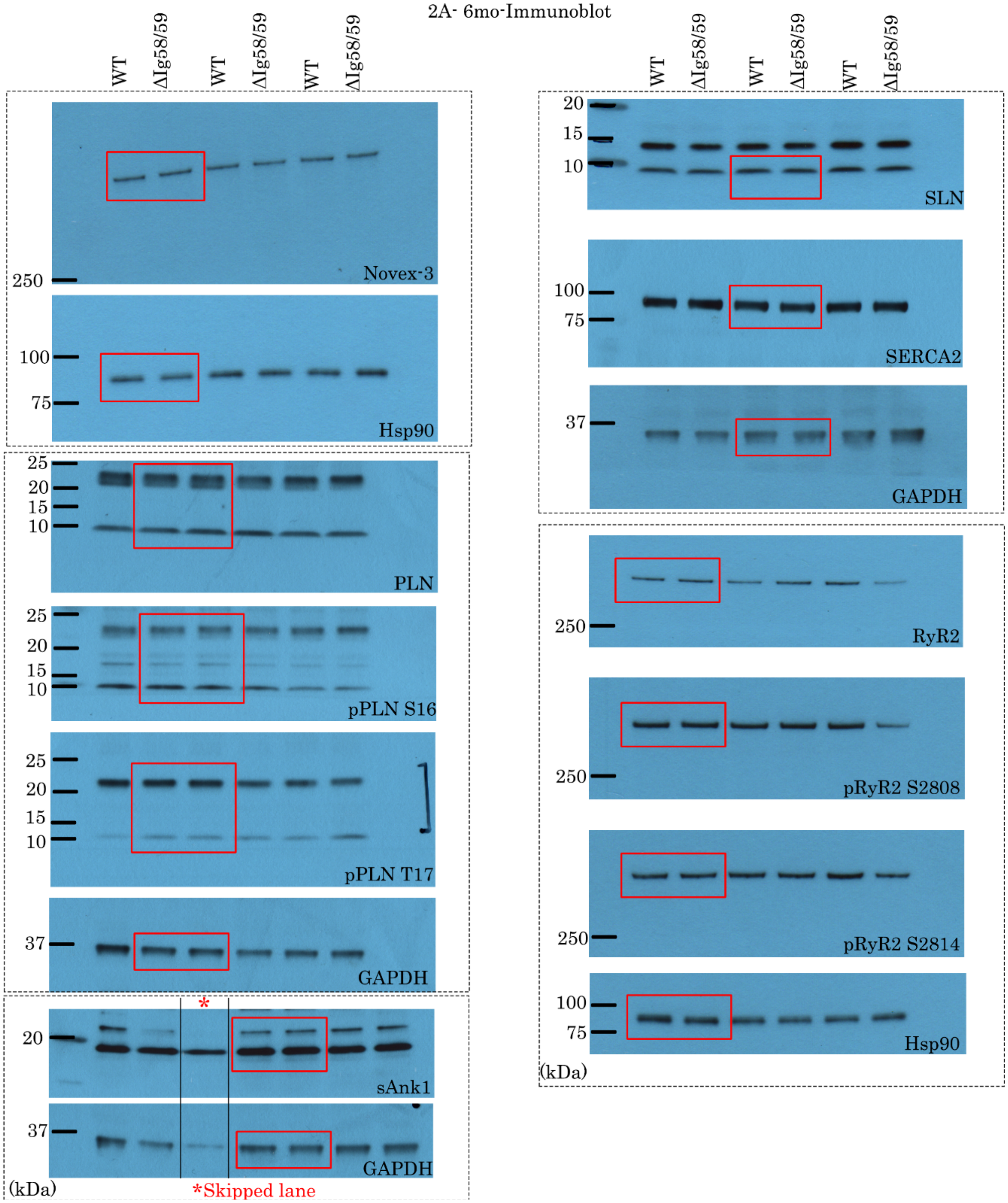

Original blots in Fig. 2A (6-month)

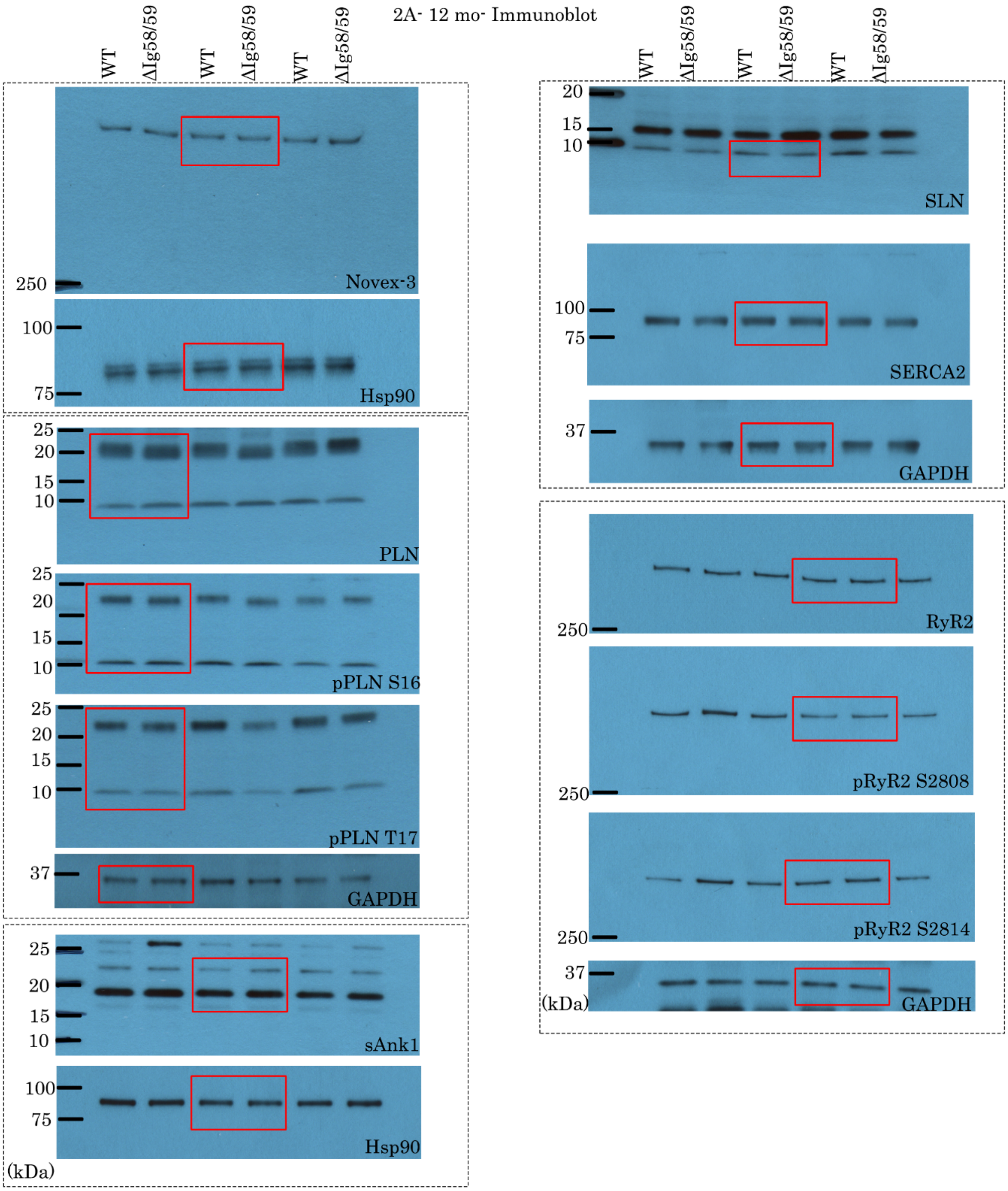

Original blots in Fig. 2A (12 month)

8A

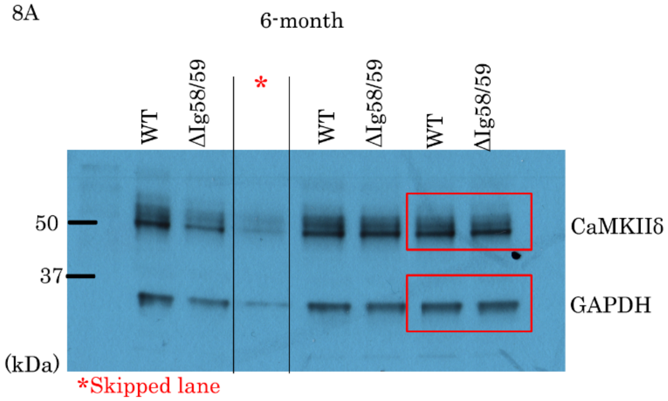

Original blots in Fig. 8A

8B

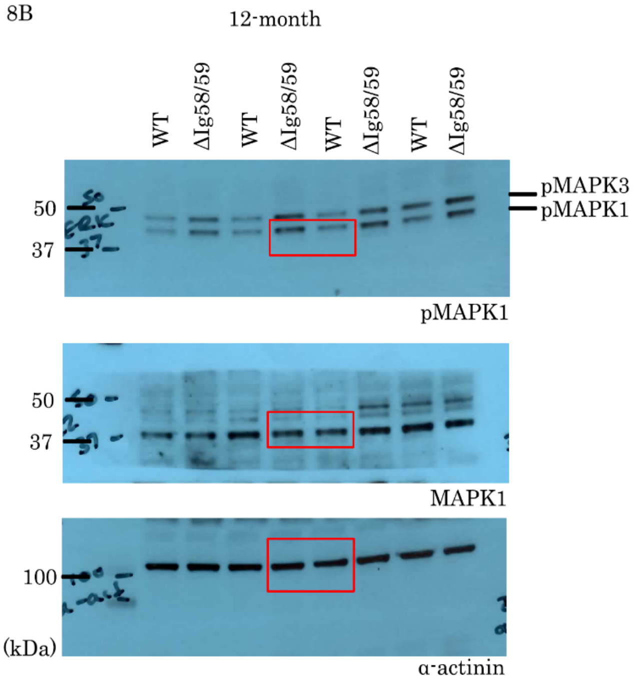

Original blots in Fig. 8B: The phospho-p44/p42 antibody (#4370; Cell Signaling) recognizes both phospho-MAPK1 (p42; also known as ERK2) and phospho-MAPK3 (p44; also known as ERK1). Phospho-MAPK3 appears to follow the same trend as phospho-MAPK1 in ΔIg58/59 atria, however since our proteomics study did not identify it as a differentially regulated kinase, we did not probe for total levels of MAPK3. Please note that Figure 8 only shows the phosphorylation levels of MAPK1 (that was identified in our proteomics screen), as illustrated by the red boxed area.
